# The RNA-binding protein Psc1 functions downstream of the NDR/LATS kinase Cbk1 to modulate CO_2_ tolerance in *Cryptococcus neoformans*

**DOI:** 10.1128/mbio.02051-26

**Published:** 2026-09-21

**Authors:** Emma E. Blackburn, Laura C. Ristow, Xiaofeng Xie, Damian J. Krysan, Xiaorong Lin

**Affiliations:** 1 Department of Microbiology, University of Georgia 1355, Athens, Georgia, USA; 2 Department of Pediatrics, Carver College of Medicine, University of Iowa 4083, Iowa City, Iowa, USA; 3 Department of Molecular Physiology and Biophysics, Carver College of Medicine, The University of Iowa 4083, Iowa City, Iowa, USA; 4 Department of Plant Biology, University of Georgia 166832, Athens, Georgia, USA; Instituto Carlos Chagas, Curitiba, Brazil

**Keywords:** *Cryptococcus neoformans*, post-transcriptional regulation, virulence, biomolecular condensates, stress adaptation, asymmetric localization

## Abstract

*Cryptococcus neoformans* is an opportunistic fungal pathogen responsible for approximately 20% of deaths in patients with HIV/AIDS. Adaptation to host physiological conditions, including high CO_2_, is required for infection. We discovered that an uncharacterized protein with an RNA-binding domain, Psc1, functions as a basidiomycete-specific suppressor of the kinase mutant *cbk1*Δ and partially rescues its growth defect in high CO_2_. Psc1 contains multiple consensus cell wall biosynthesis kinase 1 (Cbk1) phosphorylation sites that are required for maintenance of CO_2_ fitness. We hypothesized that, in the absence of Cbk1, Psc1 negatively regulates CO_2_ tolerance by binding to and interfering with the function of mRNAs required for CO_2_ tolerance. Supporting this model, we found that multiple mRNAs that are required for CO_2_ tolerance associate with Psc1 in the absence of Cbk1. Furthermore, the transcripts of *ZDS3*, a gene required for CO_2_ tolerance, predominantly colocalize with Psc1, which forms condensates in the *cbk1*Δ mutant at high CO_2_, correlating with impaired growth. Collectively, our findings support the conclusion that *C. neoformans* adapts to high CO_2_ through a post-transcriptional mechanism where Cbk1 phosphorylates Psc1, preventing its binding to, and subsequent functional inhibition of, mRNAs important for CO_2_ tolerance.

**IMPORTANCE:**

High CO_2_ is growth-inhibitory, and therefore adaptation to high CO_2_ stress is a universal cellular phenomenon required for survival. The environmental fungus, *Cryptococcus neoformans*, encounters high CO_2_ environments in the host, and thus tolerance to high CO_2_ is critical for its ability to cause lethal infections. The regulation of Ace2p and morphogenesis (RAM) pathway is required for its growth in high CO_2_. We found that CO_2_ tolerance is mediated post-transcriptionally by a downstream target of the RAM pathway, the RNA-binding protein Psc1. In the absence of the RAM pathway, Psc1 sequesters mRNA transcripts required for growth in high CO_2_, preventing growth. These findings reveal a previously uncharacterized post-transcriptional regulatory mechanism that controls cryptococcal adaptation to high CO_2_.

## INTRODUCTION

*Cryptococcus neoformans* is an environmental fungus responsible for approximately 112,000 deaths annually (1). With no vaccine available and limited effectiveness of current antifungal treatment regimens, this fungus is a deadly threat to those on immunosuppressive therapies or with compromised immune systems (e.g., recipients of organ/tissue transplants and patients with cancer or HIV/AIDS). Until recently, three major virulence traits of *C. neoformans* have been extensively investigated: growth at host body temperature, capsule formation, and melanin production. However, many strains possess all three virulence traits yet fail to replicate in murine infection models, arguing for the possibility of additional traits. Consistent with this conclusion, our group has found that the ability to grow at host concentrations of CO_2_ is a virulence trait (2). These findings highlight the drastic physiological adaptation required for this environmental fungus to proliferate within the host, where CO_2_ levels are >100-fold higher (5%–10%) than the ambient levels of CO_2_ (~0.04%) found in its natural ecological niche. It is important to note that as an environmental fungus, *C. neoformans* must be able to grow in ambient CO_2_, which requires carbonic anhydrase (3). However, carbonic anhydrases are dispensable for growth in high CO_2_, and such mutants are fully virulent in animal models (3). High CO_2_ is known to inhibit the growth of many microbes and higher eukaryotes, although different organisms tolerate varying levels of CO_2_ (4, 5). However, the molecular and cellular determinants governing adaptation to high CO_2_ conditions, particularly in the setting of infection, are incompletely understood.

We initially explored the molecular mechanisms underlying growth in high CO_2_ using large-scale genetic screens to identify mutants that affect CO_2_ tolerance (6, 7). As expected for a complex trait, we identified several major pathways critical for growth in CO_2_, including the regulation of Ace2p and morphogenesis (RAM) pathway (6, 7). Deletion mutants of any of the five RAM pathway components, including the terminal serine/threonine protein kinase Cbk1 (cell wall biosynthesis kinase 1), led to reduced CO_2_ fitness. Cbk1 belongs to the NDR/LATS kinase family and the Cbk1 homologs in higher eukaryotes are central regulators of cell proliferation, apoptosis, and morphogenesis (8–10).

In the model ascomycete yeast *Saccharomyces cerevisiae*, the RAM pathway governs multiple biological processes, including cell wall integrity, cell cycle progression, and daughter cell-specific gene expression (11–14). Loss of RAM pathway function in *S. cerevisiae* disrupts the normal asymmetric physiological state between daughter and mother cells and interferes with the proper segregation of age-accumulated cellular damage factors and proteins necessary for cell cycle progression, which impedes cell division and growth (15–17). However, it is unclear whether the downstream effectors of the RAM pathway are likely to be conserved because clear differences exist between *C. neoformans* and *S. cerevisiae*. For example, disruption of the RAM pathway in *C. neoformans* results in hyperpolarized growth (18), whereas there is a loss of polarized growth in *S. cerevisiae* (11, 14). Additionally, in *Candida albicans* and *S. cerevisiae*, Cbk1 regulates the zinc finger transcription factor Ace2, which controls daughter cell-specific gene regulation (14, 19); yet, there is no Ace2 homolog in *C. neoformans* or in any other basidiomycetes.

To understand how the RAM pathway regulates CO_2_ tolerance in *C. neoformans* and to identify its downstream effectors in basidiomycetes, we previously screened for spontaneous suppressors that rescued the growth defect of the *cbk1*Δ mutant in high CO_2_ and identified loss-of-function mutations in Ssd1 and Psc1 (6). Although mutations in both suppressors partially restored the growth defect of *cbk1*Δ in high CO_2_, neither restored the impaired transcriptome response of *cbk1*Δ to high CO_2_ (6). This observation prompted us to postulate that the RAM pathway regulates CO_2_ tolerance largely post-transcriptionally. Ssd1 is a conserved RNA-binding protein in both ascomycetes and basidiomycetes, and a known substrate of Cbk1 in *S. cerevisiae and C. albicans*. Psc1 is an uncharacterized protein containing an RNA-binding domain (RBD), one component of a poly(A)-specific ribonuclease (PARN) domain (6). The PARN domain is comprised of three domains: the CAF1 nuclease domain, the R3H non-specific RNA-binding domain, and the polyA-specific RNA-binding domain. Psc1 contains an RBD, (PF08675), which is predicted to have high specificity and affinity for poly(A) tails. We hypothesized that this potential RNA-binding protein contributes to the post-transcriptional mechanism of Cbk1 regulating CO_2_ tolerance. Therefore, we decided to explore Psc1, a novel potential downstream effector of Cbk1 in this study.

## RESULTS

### Psc1 phosphorylation contributes to *C. neoformans* CO_2_ tolerance

**Fig 1:**
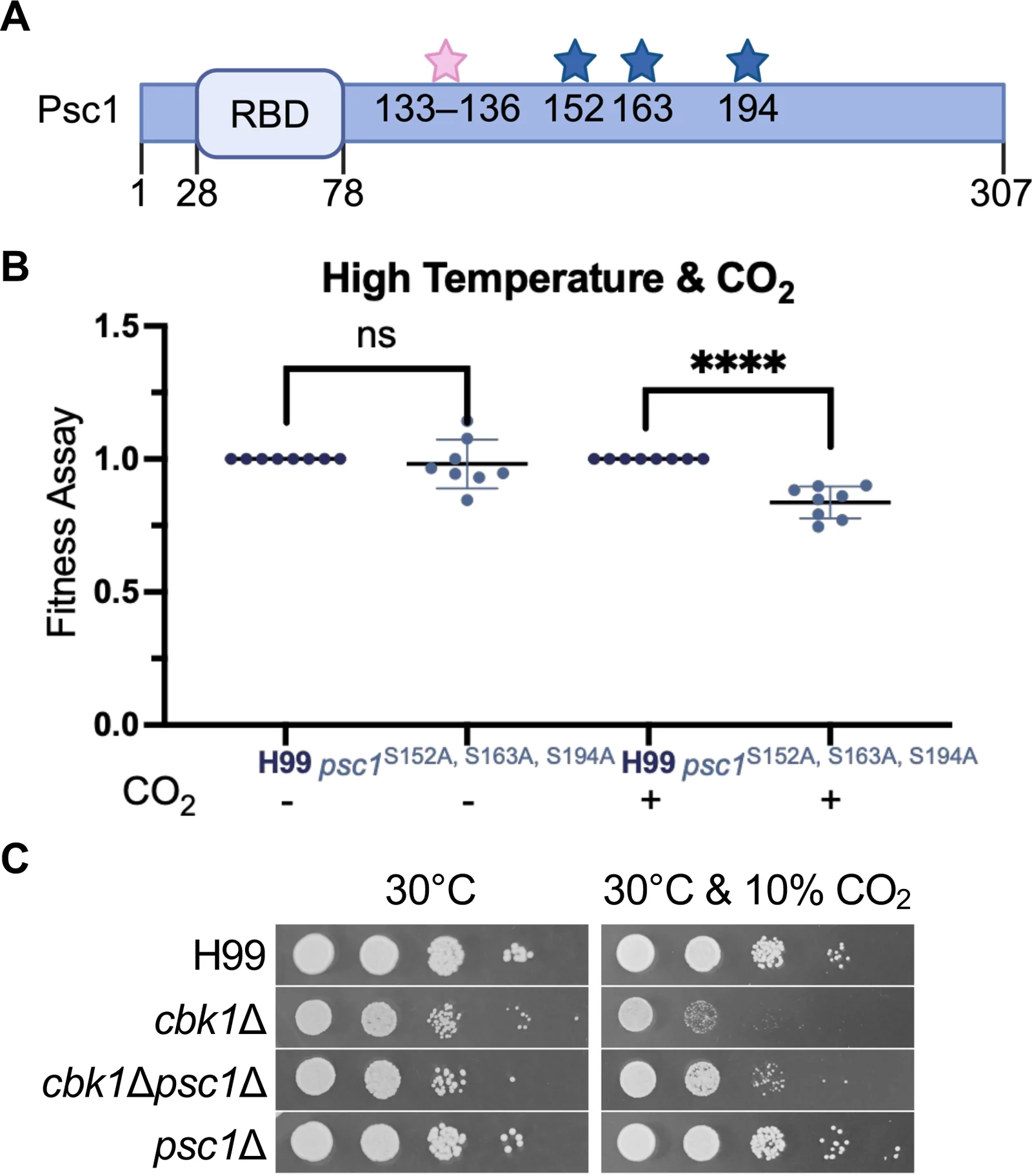
The RNA-binding protein Psc1 is a target of Cbk1. (**A**) Psc1 protein diagram including the RBD (PF08675), predicted Cbk1 phosphorylation motifs, and predicted Cbk1 docking motif. Blue stars indicate the three predicted serine residues within the Cbk1 phosphorylation motif, and the pink star indicates the predicted Cbk1 docking motif. Diagram created with BioRender (https://www.biorender.com/). (**B**) Fitness assay of wild-type H99 strain versus a strain expressing the *psc1* phospho-null allele (*psc1*^S152A, S163A, S194A^) at high temperature (37°C) and high CO_2_ (10%). An unpaired two-tailed *t*-test was performed for both conditions, graph depicts mean ± SEM, line at median (*n* = 8), *P*_37°C H99 vs 37°C *psc1*_^S152A, S163A, S194A^ = ns (0.5823), *P*_37°C and CO2 H99 vs 37°C and CO2 *psc1*_^S152A, S163A, S194A^ ≤ 0.0001 (****). (**C**) Tenfold serial dilutions from overnight cultures of wild-type H99, *cbk1*Δ, *cbk1*Δ*psc1*Δ, and *psc1*Δ strains were spotted onto YPD media and incubated for 48 h in ambient or high CO_2_ before images were acquired.

Infection-associated tissues can reach local CO_2_ concentrations of up to 10% (20). Because CO_2_-sensitive mutants such as *cbk1*Δ have modestly increased sensitivity at 10% (Fig. S1A), we used 10% CO_2_ throughout this study. To assess the function of Psc1, we began by investigating the interaction between this putative RNA-binding protein and Cbk1. Psc1 contains both Cbk1 phosphorylation and docking motifs, suggesting that Psc1 may be a direct Cbk1 substrate (Fig. 1A). The Cbk1 consensus phosphorylation motifs in Psc1, HXRXXS/T and the HXKXXS/T variant (21), are located at serine residues 152, 163, and 194. A conserved Cbk1 docking motif [F/Y]x[F/Y]P (22) is located at positions 133–136 (FPFP) within an intrinsically disordered region. The close amino acid proximity of the docking motif to the phosphorylation sites further points toward Psc1 as a potential target of Cbk1. To interrogate the potential regulation of Psc1 by Cbk1 phosphorylation, we generated a non-phosphorylatable *PSC1* allele (*psc1*^S152A, S163A, S194A^) and overexpressed the mutant allele in wild-type (WT) cells. We postulate that Cbk1-mediated phosphorylation of Psc1 contributes to CO_2_ tolerance, such that non-phosphorylated Psc1 partially mediates the CO_2_ sensitivity of the *cbk1*Δ mutant. This hypothesis predicts that overexpression of the non-phosphorylatable *PSC1* allele, even in the wild-type strain, would reduce fungal fitness in high CO_2_. Concordant with this hypothesis, overexpression of *psc1*^S152A, S163A, S194A^ modestly reduced cryptococcal fitness in high CO_2_ (Fig. 1B; Fig. S1B and C). Although neither deletion nor overexpression of the wild-type allele of *PSC1* alone in the wild-type strain affects *C. neoformans* tolerance to high CO_2_ (Fig. 1C; Fig. S1D), deletion of *PSC1* in the *cbk1*Δ mutant partially suppresses its growth phenotype in 10% CO_2_ and results in cell morphologies intermediate between round (WT-like) and pseudohyphal (*cbk1*Δ-like) cells (6) (Fig. 1C; Fig. S1E). These data are compatible with the conclusion that Psc1 is a direct target of Cbk1, and with the model that loss of Psc1 phosphorylation contributes to the CO_2_ sensitivity of the *cbk1*Δ mutant.

### Psc1 is associated with mRNAs important for CO_2_ tolerance

Because Psc1 is predicted to be an RNA-binding protein, we hypothesized that in the wild-type strain, Cbk1 phosphorylates Psc1 and thereby prevents its interaction with specific mRNA transcripts important for CO_2_ tolerance. The following genetic evidence supports this hypothesis. First, poor growth of the *cbk1*Δ mutant in high CO_2_ is partially relieved by deletion or loss-of-function mutations of *PSC1*. Second, overexpression of the *psc1* phospho-null mutant allele in wild-type cells causes a modest reduction in CO_2_ fitness in high CO_2_.

**Fig 2:**
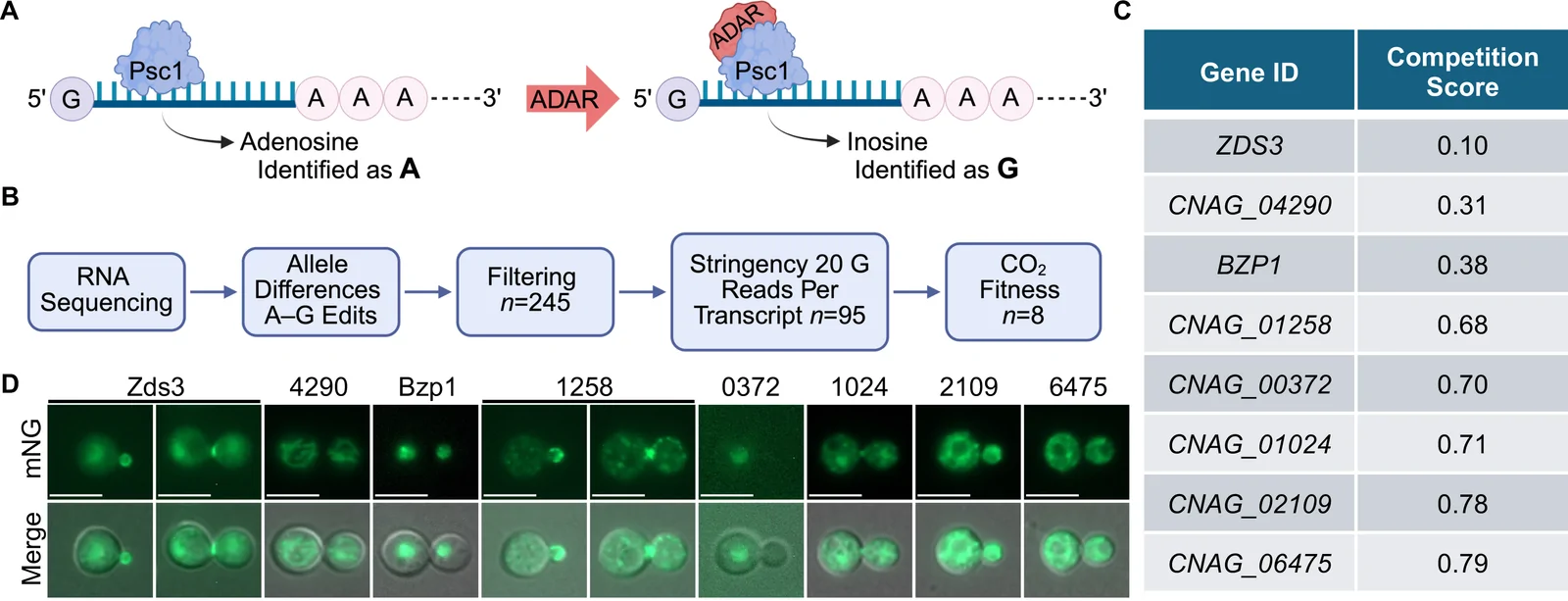
Identification of candidate mRNAs associated with the RNA-binding protein Psc1 through HyperTRIBE. (**A**) Schematic representation of the HyperTRIBE assay, created with BioRender (https://www.biorender.com/). (**B**) Schematic representation of the HyperTRIBE filtering analysis employed, created with BioRender. The full list of 95 transcripts can be found in Table S1. (**C**) Table of the filtered Psc1 mRNA candidates of interest identified with HyperTRIBE and the corresponding fitness score in high CO_2_. A score below 1.0 indicates a CO_2_-specific fitness defect. *ZDS3*, *CNAG_04290*, *BZP1*, *CNAG_01258*, *CNAG_01024*, and *CNAG_06475* had a statistically significant CO_2_ fitness defect by a Student’s *t*-test *P* < 0.05. (**D**) Images of cells expressing the eight candidate targets of Psc1 were acquired after 6 h of induction with galactose at 30°C; the scale bar indicates 5 μm.

Our hypothesis predicts that specific mRNAs bound by Psc1 in the absence of *CBK1* are important for CO_2_ tolerance. To identify the mRNA targets of Psc1, we adapted the Hyper Targets of RNA-binding proteins Identified By Editing (HyperTRIBE) method, an RNA-editing-based proximity labeling technique utilized in *S. cerevisiae*, *Ustilago maydis*, and mammalian systems to identify mRNAs associated with RNA-binding proteins (23–25). Specifically, we fused the adenosine-deaminase-acting-on-RNA catalytic domain (ADARcd) to the C-terminus of Psc1 (Fig. 2A). When Psc1-ADARcd binds to or interacts with an mRNA transcript, the ADARcd domain catalyzes the deamination of adenosine to inosine. Inosine is detected as guanosine during high-throughput RNA sequencing and, therefore, marks transcripts that are associated with Psc1 (Fig. 2A). To ensure that the addition of ADARcd to Psc1 did not disrupt its function prior to the HyperTRIBE experiment, we fused an mNeonGreen (mNG) fluorescent reporter to Psc1-ADARcd. We observed that Psc1-ADARcd-mNG was expressed, and its expression restored the *cbk1*Δ*psc1*Δ mutant to the single *cbk1*Δ mutant cell morphology (Fig. S2A). These results indicate that fusing ADARcd to Psc1 does not disrupt its function.

For the HyperTRIBE experiment, we leveraged the tightly controlled inducible *GAL10* promoter system to regulate expression of Psc1-ADARcd in the *cbk1*Δ*psc1*Δ mutant strain. Psc1-ADARcd is functional because it restores the *cbk1*Δ*psc1*Δ double-mutant phenotype to that of the *cbk1*Δ single-mutant phenotype upon induction (Fig. S1E). Expression of Psc1-ADARcd was induced by incubation in galactose-containing media for 3 h; the cells were then shifted to 10% CO_2_ for two additional hours in galactose. As a control, the same HyperTRIBE-containing strains were incubated in non-inducing, dextrose-containing media under otherwise identical conditions. Cells from the inducing and non-inducing conditions were harvested, RNA was extracted, and mRNAs were sequenced (sequencing depth 139× to 185×) to identify transcripts with adenosine-to-guanosine (A–G) editing.

The sequencing data were processed using previously reported HyperTRIBE analysis pipelines (23–25). After applying our selection criteria as described in Materials and Methods, we identified 95 high-confidence A–G edited transcripts (Fig. 2B and Table S1). Of these 95 genes, those with predicted functions in stress response (e.g., high temperature, chemical, reactive oxygen species) and cell wall organization or cell wall biosynthesis are consistent with our hypothesis because these transcripts are important for growth in high CO_2_. To prioritize transcript candidates important for CO_2_ tolerance, we tested the fitness of their gene deletion mutants (reflecting the loss of these transcripts) under high CO_2_ conditions. We compared the set of 95 genes to our recently reported large-scale screen for mutants with altered fitness at 5% CO_2_ (26). Twenty-one genes were not included in the partial genome deletion collection. Of the 74 candidate Psc1 targets, eight had a CO_2_ fitness defect ranging from 0.10 to 0.79 (Fig. 2C), with six being statistically significant, providing orthogonal evidence connecting these genes in CO_2_-related processes. Additionally, we examined published phenotypic landscape profiling data from the Madhani Laboratory (27). Of the six genes with statistically significant growth defects at high CO_2_, four also exhibited statistically significant defects in virulence in an *in vivo* mouse fitness assay (*ZDS3*, *BZP1*, *CNAG_01258*, and *CNAG_06475*; no data were available for *CNAG_04290*), suggesting that several identified genes may contribute to cryptococcal fitness *in vivo*.

To examine the potential role of these eight candidates, we first assessed their subcellular localization (Fig. 2D). To that end, we fused an mNeonGreen fluorescent reporter to their C-terminus and drove their expression using the inducible *GAL10* promoter. Three of the candidates (Bzp1, CNAG_00372, and Zds3) localized to the nucleus (28) (Fig. 2D; Fig. S2B). Two of the candidates (CNAG_02109 and CNAG_06475) showed perinuclear localization (Fig. 2D; Fig. S2C), consistent with the endoplasmic reticulum (ER). These two proteins are likely involved in ER stress response and membrane function. Three of the candidates displayed striking and dynamic localization patterns: all three showed transient localization to the septum (Zds3, CNAG_01258, and CNAG_01024) during cell division, and two were enriched in daughter cells (Zds3 and CNAG_01258). In model yeast, Zds proteins are cell-cycle-regulated and play important roles in cell polarity, cell morphology, and cell wall integrity (29–31). Therefore, we prioritized *ZDS3* because it localizes to the sites of expected Cbk1 function (i.e., polarized growth and cell division) and because the *zds3*Δ mutant has a profound fitness defect in high CO_2_ (Fig. 2C and D and Table S1).

### Overexpression of *ZDS3* can rescue the morphological defect of *cbk1*Δ and partially alleviate its growth defect

**Fig 3:**
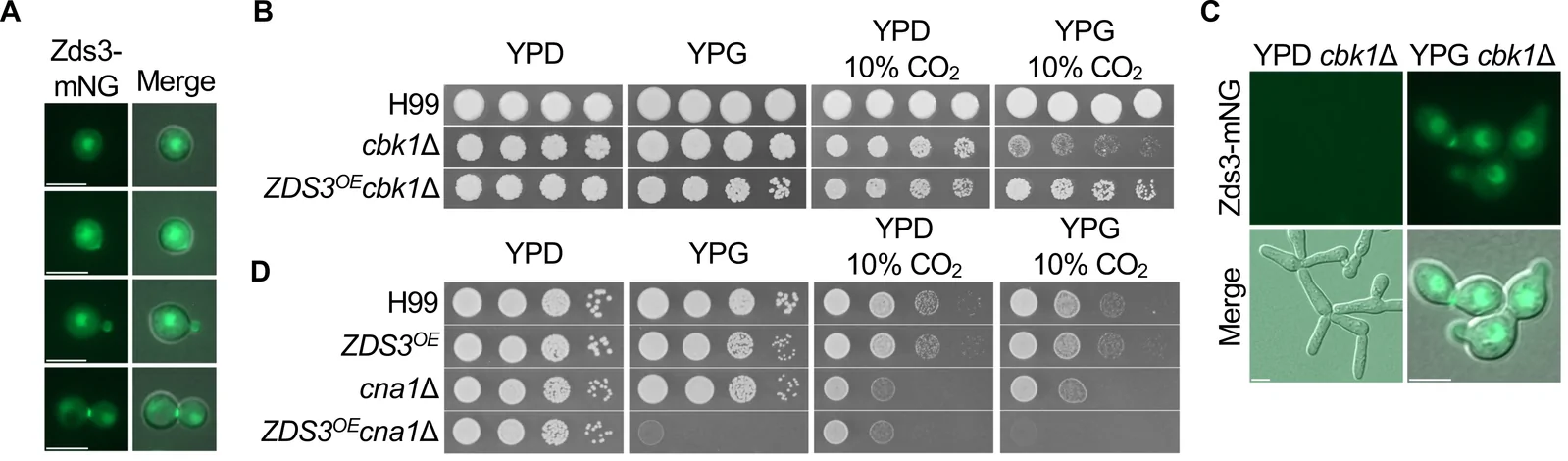
Overexpression of *ZDS3* partially restores the defects of the *cbk1*Δ mutant but not the *cna1*Δ mutant. (**A**) Images of cells expressing Zds3-mNG were acquired after 6 h of induction with galactose at 30°C; the scale bar indicates 5 μm. (**B**) Serial dilutions (2.5-fold) from overnight cultures of wild-type H99, *cbk1*Δ, and *ZDS3*^OE^*cbk1*Δ were spotted onto YPD (*ZDS3* non-inducing) and YPG (*ZDS3* inducing) media and incubated for 72 h in ambient CO_2_ or high CO_2_ before images were acquired. (**C**) Images were taken after 8 h of induction with galactose or 8 h of non-inducing conditions, both at 30°C; the scale bar indicates 5 μm. (**D**) Tenfold serial dilutions from overnight cultures of wild-type H99, *ZDS3*^OE^, *cna1*Δ, and *ZDS3*^OE^*cna1*Δ were spotted onto YPD (*ZDS3* non-inducing) and YPG (*ZDS3* inducing) media and incubated for 48 h in ambient CO_2_ or high CO_2_ before images were acquired.

To further characterize the Psc1 candidate target *ZDS3*, we first examined its subcellular localization using an mNeonGreen fluorescent reporter fused to the C-terminus of Zds3, driving its expression by the inducible *GAL10* promoter. When introduced into the z*ds3*Δ mutant, Zds3-mNG complemented the temperature- and CO_2_-sensitive phenotypes, indicating that the Zds3-mNG fusion is functional (Fig. S3A and B). The Zds3 protein exhibited dynamic cell-cycle-dependent subcellular localization (Fig. 3A). In non-budding cells, Zds3 localized to the nucleus. In cells with an emerging budding site, Zds3 accumulated at the incipient bud site in addition to the nucleus. Initially, Zds3 localized increasingly at the daughter cortex. During isotropic growth, Zds3 localized to the septum prior to cytokinesis. Taken together, the cell-cycle-dependent subcellular localization of Zds3 and the reduced fitness of the *zds3*Δ mutant in high CO_2_ indicate its potential significance as a factor required for growth in CO_2_.

We posited that forced overexpression of Cbk1/Psc1-regulated mRNAs important for growth in high CO_2_, such as *ZDS3*, in the *cbk1*Δ mutant may overwhelm the binding capacity of unregulated Psc1. This could partially alleviate the deleterious effects of unregulated Psc1-mediated mRNA binding/sequestration, and thereby partially restore growth of the *cbk1*Δ mutant in high CO_2_. Overexpression of *ZDS3* indeed modestly rescued the CO_2_ growth defect of the *cbk1*Δ mutant (Fig. 3B). In addition, *ZDS3* overexpression completely restored the pseudohyphal morphotype of the *cbk1*Δ mutant to a round-yeast morphology, while the cell separation defect remained evident (Fig. 3C). Notably, overexpression of *ZDS3* in wild-type cells conferred no growth advantage nor alteration of yeast morphotype (Fig. S3A and B). To assess whether the phenotypic changes caused by *ZDS3* overexpression were consistent within mutants of the RAM pathway, we also tested the effect of *ZDS3* overexpression in the *mob2*Δ mutant. In model yeast, Mob2 binds to Cbk1 and is required for its function (13, 32). As expected, overexpression of *ZDS3* partially restored growth of the *mob2*Δ mutant in high CO_2_ (Fig. S3C), and completely restored the pseudohyphal morphotype of the *mob2*Δ mutant to a round-yeast morphology (Fig. S3D). To ascertain whether *ZDS3* overexpression is a general suppressor of mutants with reduced CO_2_ tolerance, we tested its effect on the *cna1*Δ mutant, which lacks the catalytic subunit of the phosphatase calcineurin. We previously found calcineurin to be critical for CO_2_ tolerance (Fig. 3D [6]). To our surprise, instead of mitigating some of the *cna1*Δ mutant’s growth defects, overexpression of *ZDS3* in the *cna1*Δ mutant caused a profound growth defect in ambient CO_2_ and was synthetically lethal in high CO_2_ (Fig. 3D). Thus, overexpression of *ZDS3* does not confer a general growth advantage to strains with CO_2_ fitness defects. Rather, it demonstrates some level of specificity to the RAM pathway.

Collectively, the modest restoration of growth in high CO_2_ and the marked rescue of the *cbk1*Δ mutant cell morphology by *ZDS3* overexpression align with a role for *ZDS3* in CO_2_ tolerance and provide a functional connection between this Psc1 target mRNA and the RAM pathway. These findings are consistent with a model in which Cbk1 phosphorylates Psc1, preventing its binding to mRNAs, such as *ZDS3*, and supports growth in high CO_2_.

### Psc1 forms foci and is enriched in daughter cells in response to CO_2_

**Fig 4:**
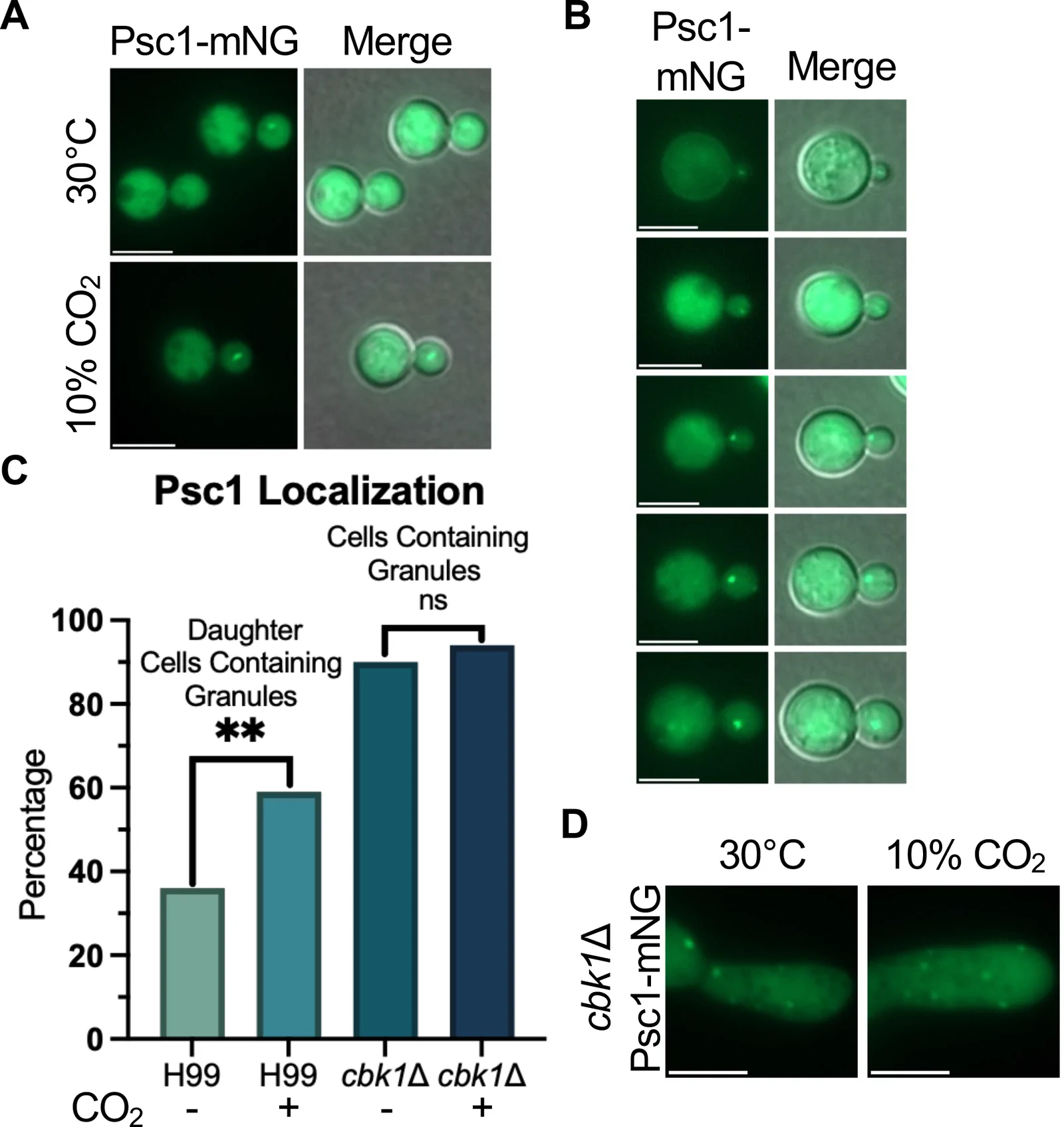
Psc1 forms foci that are enriched in the daughter cells in response to CO_2_. (**A, B, and D**) The scale bar indicates 5 μm. Images were captured after 6 h of induction on YPD with bathocuproinedisulfonic (BCS) acid; the first 4 h at 30°C, followed by a 2-h shift to 10% CO_2_ for the high CO_2_ condition, and for ambient CO_2_, the cells remained at 30°C for two additional hours. (**A**) Psc1-mNG localization in the wild-type strain at 30°C in ambient and 10% CO_2_. (**B**) Psc1-mNG localizes to daughter cells irrespective of daughter cell size. (**C**) At least three independent experimental replicates were used for quantification. A two-sided Fisher’s exact test was performed. For the wild-type strain, at least 72 budding cells were quantified for each condition. *n*_30°C_ = 72 and *n*_CO2_ = 78. *P*_30°C vs CO2_ = 0.0057 (**). For *cbk1*Δ, at least 63 cells were quantified for each condition. *n*_30°C_ = 63 and *n*_CO2_ = 65. *P*_30°C vs CO2_ = ns (0.4396). (**D**) Psc1-mNG localizes to distinct foci in the *cbk1*Δ mutant.

To further investigate the relationship between Psc1 protein and *ZDS3* transcripts, we visualized both with fluorescence microscopy. First, we fused an mNeonGreen fluorescent reporter to the C-terminus of Psc1 under the control of an inducible promoter (*CTR4* or *GAL10*). We confirmed that the Psc1-mNG fusion is functional through complementation, as its introduction into the *cbk1*Δ*psc1*Δ mutant restores the double-mutant phenotype to that of the *cbk1*Δ single-mutant upon expression (Fig. S1E). At 30°C, Psc1-mNG expressed in the wild-type strain displayed diffuse cytosolic localization in both mother and daughter cells (Fig. 4A). Interestingly, Psc1 formed distinct foci that localized asymmetrically to daughter cells irrespective of daughter cell size (Fig. 4B). These distinct foci formed in 36% of the daughter cells in ambient CO_2_ and increased to 59% in high CO_2_ (Fig. 4C).

To test whether this asymmetric localization and foci formation were the result of overexpression, we constructed Psc1-mNG driven by its native promoter. The native promoter-driven Psc1-mNG also displayed asymmetric localization in distinct foci in the daughter cell (Fig. S4A and B). These discrete foci were observed in 33% of daughter cells at 30°C and 57% at 10% CO_2_ (Fig. S4C). These foci were similar in shape (spherical or ovoid) and variable in size (Fig. S4D). Thus, localization of Psc1 was similar in strains in which it was expressed from either the native promoter or the inducible, heterologous promoter.

The localization of Psc1-mNG driven by either an inducible promoter or the native promoter in the *cbk1*Δ mutant exhibited foci dispersed throughout the cytoplasm (Fig. 4D; Fig. S4E and F). Specifically, ~90% of pseudohyphae contained Psc1 foci in either ambient CO_2_ or high CO_2_ (Fig. 4C; Fig. S4E). These findings demonstrate that Psc1 localizes to well-demarcated foci in a Cbk1-independent manner. However, the asymmetric localization of these foci to daughter cells is only apparent with functional Cbk1.

### Psc1 colocalizes with *ZDS3* transcripts in the absence of *CBK1*

**Fig 5:**
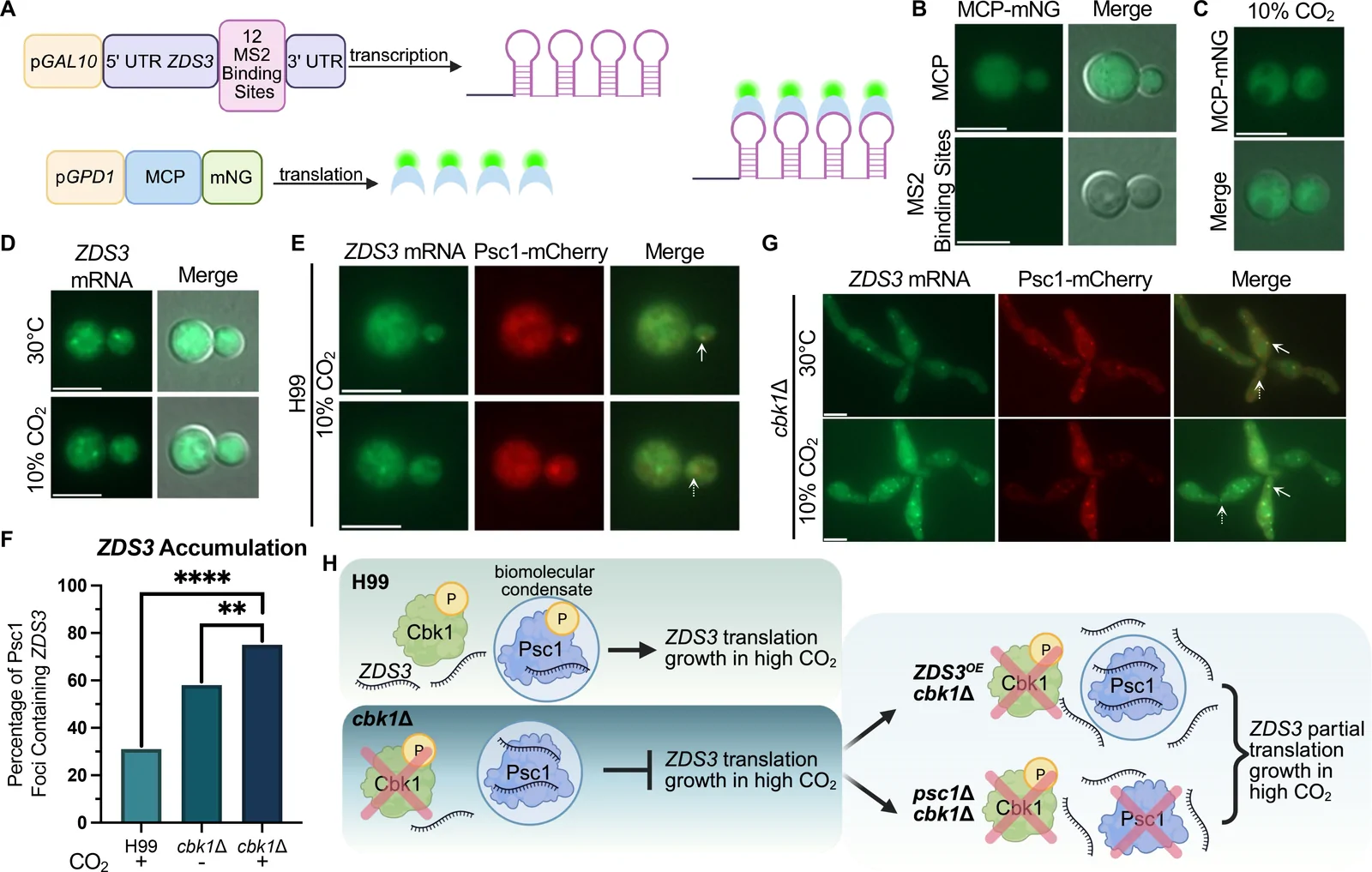
Psc1 sequesters *ZDS3* transcripts in the absence of *CBK1* and inhibits growth in CO_2_. (**A**) Schematic representation of the MS2-MCP system, created with BioRender (https://www.biorender.com/). (**B–E and G**) The scale bar indicates 5 μm. (**B**) Induced expression of *ZDS3* with MS2-binding sites alone does not result in any fluorescence. Constitutive expression of MCP-mNG alone results in diffuse fluorescence throughout the cell in ambient CO_2_. (**C**) Constitutive expression of MCP-mNG alone results in diffuse fluorescence throughout the cell in high CO_2_. (**D**) Employment of the complete MS2 system (*ZDS3*-MS2 + MCP mNG) shows localization of *ZDS3* mRNA (concentrated green fluorescence) in both mother and daughter cells in ambient CO_2_ and high CO_2_. (**E**) Visualization of *ZDS3* mRNA (mNG) with Psc1 protein (mCherry) in wild-type H99 cells. (**E and G**) Solid arrows indicate colocalization, and dashed arrows indicate no colocalization of *ZDS3* transcripts and Psc1 protein. Images were captured after 6 h of induction with galactose; 4 h at 30°C, followed by a 2-hour shift to 10% CO_2_ for the high CO_2_ condition, and for the ambient CO_2_, the cells remained at 30°C for two additional hours. (**F**) A two-sided Fisher’s exact test was performed. At least three independent experimental replicates were used for quantification. At least 148 Psc1 foci were quantified, *n*_30°C *cbk1*Δ_ = 154, *n*_CO2 *cbk1*Δ_ = 148, and *n*_30°C H99_ = 153, *P*_30°C *cbk1*Δ vs CO2 *cbk1*Δ_ = 0.0023 (**); *P*_CO2 *cbk1*Δ vs CO2 H99_ ≤ 0.0001 (****). (**G**) Visualization of *ZDS3* mRNA (mNG) with Psc1 protein (mCherry) in *cbk1*Δ cells. (**H**) Model for the post-transcriptional role of Psc1 regulated by Cbk1-dependent phosphorylation, created with BioRender.

We hypothesized that, in the wild-type strain, Cbk1 phosphorylation of Psc1 prevents it from interacting with *ZDS3* transcripts and allows it to be translated into functional proteins. In the absence of Cbk1 regulation, however, Psc1 binds to *ZDS3* transcripts, leading to reduced *ZDS3* function. Thus, we would expect Psc1 protein and *ZDS3* transcripts to colocalize when Cbk1 function is disrupted. To test this hypothesis, we employed the MS2-MS2 coat protein (MCP) tagging system to visualize the localization of *ZDS3* transcripts (33). This is a two-component system that utilizes the ability of the MCP to specifically recognize and bind to the MS2-binding sites (MBS) (Fig. 5A). Here, we added 12 MBS sequences following the *ZDS3* stop codon within the 3′ untranslated region and drove its expression using the inducible *GAL10* promoter. The addition of the MBS alone yielded no detectable fluorescence (Fig. 5A and B). In parallel, we fused an mNG reporter to the MCP and drove its expression using the constitutive *GPD1* promoter, which expectedly displayed diffuse cytosolic localization under all conditions tested, including at 30°C in ambient CO_2_ or with 10% CO_2_ (Fig. 5A through C). When both constructs are present in the same cell, interaction between the MCP and MBS results in a concentrated fluorescence signal, allowing direct visualization of the labeled mRNA transcripts (Fig. 5A). Employing this system, we observed *ZDS3* transcripts in both mother and daughter cells at 30°C in both ambient and high CO_2_ conditions in the wild-type strain (Fig. 5D).

Now, with the strain harboring both Psc1-mCherry and mNG-MCP-*ZDS3*-MS2, we examined their localization pattern with functional Cbk1. We observed that 31% of Psc1 foci contained *ZDS3* transcripts in response to high CO_2_ (Fig. 5E and F). Next, we asked whether Cbk1 had an effect on the colocalization of *ZDS3* mRNA and Psc1 protein. In the absence of Cbk1, 57% of Psc1 foci colocalized with *ZDS3* transcripts in ambient CO_2_, which increased to 75% in high CO_2_ (Fig. 5F and G). These data indicate that only a minority of *ZDS3* mRNAs are bound by Psc1 when Cbk1 is functional. However, in the absence of Cbk1 and particularly also in high CO_2_ conditions, the majority of *ZDS3* transcripts are bound by dysregulated Psc1. Collectively, our findings are consistent with the hypothesis that Cbk1 phosphorylates Psc1, which likely reduces the affinity of Psc1 for *ZDS3* transcripts and permits their translation into proteins required for cryptococcal adaptation to high CO_2_ (Fig. 5H).

## DISCUSSION

In this work, we have established that the NDR/LATS kinase Cbk1 regulates the function of the putative mRNA-binding protein Psc1 as part of its role in maintaining CO_2_ tolerance. We have also illustrated two key similarities between Cbk1 regulation in basidiomycetes and ascomycetes. First, Cbk1 regulates the function of two RNA-binding proteins, Psc1 and Ssd1; second, Cbk1 regulates mother-daughter cell asymmetry in both basidiomycetes and ascomycetes.

Our recent discoveries point to the critical importance of post-transcriptional regulation in CO_2_ tolerance. We found that the cryptococcal transcriptome exhibits minimal changes after 4 h of exposure to high CO_2_. After 24 h of high CO_2_ exposure, activation of the TOR pathway, which is required for CO_2_ tolerance, results in strong upregulation of ribosome biogenesis and RNA-processing genes (7). These data further indicate that the regulation of mRNA-related processes plays an important role in CO_2_ tolerance. The results reported here provide the first evidence elucidating how Psc1 acts downstream of the critical and conserved NDR/LATS kinase Cbk1 to control the potential translation of transcripts important for CO_2_ tolerance.

Two of the primary targets of the RAM pathway kinase, Cbk1, in *S. cerevisiae* are the RNA-binding protein Ssd1 and the daughter-cell-specific transcription factor Ace2. In *S. cerevisiae,* Ssd1 is a translational repressor that is negatively regulated by Cbk1 (34–36). Cbk1 regulation of Ssd1 permits cell wall remodeling and polarized growth in *S. cerevisiae* (34–36), and herein, we demonstrated that Cbk1-dependent phosphorylation of Psc1 prevents its binding to *ZDS3*, thereby promoting CO_2_ tolerance in *C. neoformans*. In both organisms, Cbk1 regulates two different RNA-binding proteins, allowing for growth and adaptation.

Additionally, in *S. cerevisiae,* the daughter-cell-specific transcription factor Ace2, another target of Cbk1, controls septum degradation and cell separation (11, 12, 14). The septum forms a selective barrier that regulates and maintains the distinct physiological states of the physically connected mother and daughter cells. Disruption of cell asymmetry broadly impairs daughter cell growth and could result in aberrant cell signaling, premature aging, and altered homeostasis (15–17). Proper asymmetrical localization and control are critical. One way this could be achieved is through post-transcriptional regulation of the localization and translation of mRNAs important for septum formation and daughter-cell-specific factors.

As observed in *S. cerevisiae*, asymmetric distribution of proteins permits daughter- or mother-cell-specific physiology (37, 38). Ace2 activates *ASH1* expression among many other genes in daughter cells (39, 40). In *S. cerevisiae*, *ASH1* mRNA is actively transported to the bud tip, where it is translated in daughter cells, and the resulting Ash1 protein prevents expression of target genes (39, 40). No sequence or functional equivalent of Ace2 in *C. neoformans* has been identified, based on phenotypic analyses of the transcription factor deletion set and the larger partial genome deletion set. Interestingly, we observed that in *C. neoformans,* Psc1 forms foci in response to high CO_2_ and is enriched in the daughter cells (Fig. 4A through C; Fig. S4A through D). Furthermore, we found two transcripts, *ZDS3* and *CNAG_01258*, identified as targets of Psc1, encode proteins also enriched in the daughter cell and septum in *C. neoformans*. Neither *PSC1* nor *ZDS3* transcripts are cell-cycle-regulated, and among the prioritized targets, only *CNAG_02109* was identified as cell-cycle-regulated (41). This further supports that the localization patterns observed are likely post-translationally regulated. To our knowledge, Psc1 and its targets characterized here are the only proteins in *C. neoformans* documented to have daughter-cell-specific localization patterns. To date, control of expression, translation, or mRNA and protein localization in the daughter cell has remained unexplored in this basidiomycete.

Herein, we have illustrated a critical regulatory circuit governing CO_2_ tolerance in which the downstream effector of the RAM pathway, Psc1, regulates Zds3 in a post-transcriptional manner. We have only begun to understand the post-transcriptional landscape that shapes adaptation to changing environmental conditions and supports growth in host CO_2_ conditions. Based on the evidence presented in this study, we propose a model in which Cbk1-dependent phosphorylation of Psc1 prevents its sequestration of *ZDS3* mRNA, thereby enabling translation of Zds3, which is necessary for growth in CO_2_ (Fig. 5H). These observations reveal a regulatory mechanism controlling cryptococcal adaptation to high CO_2_, a key virulence trait.

## MATERIALS AND METHODS

### Strains and culture conditions

*C. neoformans* strains used are listed in Table S2. Stocked strains were maintained in 23% (vol/vol) glycerol stocks at −80°C. Prior to experimentation, strains were recovered on solid YPD (1% [wt/vol] yeast extract, 2% [wt/vol] Bacto-Peptone, 2% [wt/vol] dextrose, and 2% [wt/vol] agar) or YPD with 100 μM copper for *CTR4*-inducible strains and incubated at 30°C for 2 days.

### *C. neoformans* mutant strain constructions

To disrupt the gene *CBK1* (CNAG_03567), a nourseothricin (NAT) resistance cassette was inserted into the ORF using the transient CRISPR/Cas9 (TRACE) approach previously described (42). To generate the single-guide RNA (sgRNA) for specific targeting to the *CBK1* locus, the U6 promoter and sgRNA scaffold were amplified from JEC21 genomic DNA and the plasmid pDD162 using primers Linlab8639 and Linlab4627, as well as Linlab8640 and Linlab4628. The U6 promoter and sgRNA scaffold were fused together by overlap PCR with primers Linlab4594 and Linlab4595 to generate the final sgRNA construct. All oligonucleotides used in this study are listed in Table S4.

For plasmid construction, either T4 cloning through restriction enzyme digestion or T5 cloning was used; all plasmids used in this study are listed in Table S3. Specific primers and cloning schemes are reported in Table S4. For the MS2 hairpin construct, the MS2 hairpin was synthesized by GenScript, and a linear sequence with *ZDS3* was generated using overlap PCR. The fusion product consisted of NAT-P*_GAL10_*-5′ UTR-*ZDS3*-12 MS2-binding sites-3′ UTR-T*_GPD1_*. For the Psc1 phospho-null mutant allele (*psc1*^S152A, S163A, S194A^), two sets of primers were used (LinLab11018 and LinLab11019 S194) and (LinLab11319 and LinLab11320 S152 and S163) to create the serine-to-alanine mutations of the three residues. All overexpression constructs were amplified from the plasmids using M13 forward and reverse primers before transformation.

To generate the strain to visualize *ZDS3* (CNAG_05791) mRNA and Psc1 (CNAG_01919) protein with the *CBK1* gene deleted, the strain containing *CTR4*-PSC1-mCherry-G418 was transformed with TRACE to integrate P*_GPD1_*-MCP-mNeonGreen-HYG. This strain, containing both P*_CTR4_*-PSC1-mCherry-G418 and P*_GPD1_*-MCP-mNeonGreen-HYG, was transformed with TRACE, and the P*_GAL10_*-5′ UTR-ZDS3-12 MS2-binding sites-3′ UTR-NAT linear assembly was targeted to the *CBK1* locus to disrupt the gene.

Whole-plasmid sequencing was used to confirm that the Psc1 phospho-null mutant allele and the linear fusion product of NAT-P*_GAL10_*-5′ UTR-*ZDS3*-12 MS2-binding sites-3′ UTR-T*_GPD1_* sequences were correct prior to cryptococcal transformation.

Deletion and overexpression constructs were transformed into the recipient *C. neoformans* genome by the TRACE method as described previously (42).

Transformants were selected on YPD medium with 100 µg/mL of NAT, 100 µg/mL of geneticin (G418), or 200 µg/mL of hygromycin (HYG) depending on the marker used. The genotype of cryptococcal transformants was validated as previously described (42).

### Phospho-null allele fitness assay

Wild-type and mutant cells (1.25 × 10^6^ of each strain) were inoculated into YPG (inducing) at a 1:1 ratio and mixed. Cells were grown overnight statically at 37°C or 37°C and 10% CO_2_. Cells were collected by centrifugation, washed twice with ddH_2_O, diluted, and plated onto YPD and YPD media with 100 µg/mL of geneticin (G418) or visualized for fluorescence. Colonies were counted after 48 h of incubation at 30°C. The fitness score was calculated by dividing the colony counts from the G418 selection plate by half of the non-selective YPD plate counts. An unpaired two-tailed *t*-test was performed to assess for statistical significance. Cells were quantified with microscopy (fluorescence *psc1*^S152A, S163A, S194A^-mNG or no fluorescence H99). Fitness was calculated by dividing the fluorescent cell counts by half of the total cell counts (fluorescent and non-fluorescent). An unpaired two-tailed *t*-test was performed for statistical significance.

### Spot dilution assays

Cells from overnight cultures were washed with ddH_2_O and diluted to the cell density of OD_600_ = 1.00 using spectrophotometric measurements, and serially diluted in fold increments (increment listed in figure legend) before spotting (3 μL of each dilution) onto the indicated media. CO_2_ levels were controlled by a VWR CO_2_ incubator or by a Pro-CO_2_ controller (Biospherix, Lacona, NY, USA). Plates were incubated for the indicated time listed in the figure legend. For strains with *GAL10*-driven expression, 3 μL from each dilution was spotted onto either YPD (non-inducing) or YPG (inducing) media for each tested condition. Images were captured using the Sony E PZ 16–50 mm F3.5–5.6 OSS lens.

### Microscopy

A Zeiss Imager M2 microscope equipped with an AxioCam506 camera was used for all microscopy. A Zeiss 63× apochromat oil-immersion objective with a numerical aperture of 1.4 was used for imaging. All images were acquired and processed using ZEN Pro software. Zeiss Filter Set 38 High Efficiency (HE) was used for mNeonGreen imaging, Filter Set 63 HE was used for mCherry, and Filter Set 49 was used for DAPI. Cells were grown on fresh solid medium (either YPD for *GAL10*-driven expression or YPD with 100 μM copper for *CTR4*-driven expression) at 30°C overnight. Cells were harvested and washed twice with PBS. Cells were transferred to either YPG (1% [wt/vol] yeast extract, 2% [wt/vol] Bacto-Peptone, and 2% [wt/vol] galactose) for *GAL10*-driven expression or YPD with 100 μM BCS acid for *CTR4*-driven expression for induction. For non-inducing controls, cells were transferred to fresh medium (YPD or YPD with 100 μM copper). Cells were harvested and washed twice with 1× PBS, and the cells were imaged. Specific induction times and CO_2_ conditions are listed in the corresponding figure legends and text.

For candidate proteins with daughter-cell-specific localization, we also examined their localization under different inducing conditions. We induced expression with only 1% (wt/vol) galactose (rather than 2% [wt/vol]) and examined their distribution after only 2 h of induction to observe the earliest protein localization. In these conditions tested, brighter fluorescence appeared in daughter cells before appearing elsewhere intracellularly.

### RNA extraction

Strains were induced for 3 h on solid YPG (inducing) and shifted to 10% CO_2_ for two additional hours; control strains were treated the same, except maintained on YPD (non-inducing). Cells were washed twice with ddH_2_O, flash-frozen in liquid nitrogen, and lyophilized at −83°C under a vacuum of 0.008 mbar for 48 h. Biological triplicates were harvested for each condition (induced and non-induced). Total RNA was isolated from lyophilized cells following the manufacturer’s protocol using the PureLink RNA mini-kit, followed by DNase treatment using the Turbo DNA-free kit. Quality control was performed on extracted RNA using Qubit and agarose gel electrophoresis prior to submission for sequencing. Total RNA was submitted to Novogene for mRNA sequencing on the Illumina NovaSeq X platform, and RNA integrity was assayed with ScreenTape.

### HyperTRIBE analysis

The RNA sequencing libraries were prepared using the manufacturer’s instructions for the VAHTS Universal V8 RNA-seq Library Prep Kit for Illumina (NR605, Vazyme, Nanjing, China). Following RNA sequencing, quality control was performed via adaptor and low-quality read trimming, and the remaining reads were aligned to the H99 reference genome (FungiDB version Release 59). Both induced and uninduced RNA sequencing results were analyzed to account for promiscuous A–G editing. We compared three samples of P*_GAL10_*-Psc1-ADARcd *cbk1*Δ*psc1*Δ uninduced (without galactose) with the two samples of H99 (WT as the reference). In comparison, we identified three potential A–G editing events (average read depth ≥ 20 and the G read percentage G/[A + G] difference ≥10% in comparison to the reference). These three events were filtered out when we applied the same criteria to identify true A–G editing events in the three samples of P*_GAL10_*-Psc1-ADARcd *cbk1*Δ*psc1*Δ induced (with galactose) compared with three samples of P*_GAL10_*-Psc1-ADARcd *cbk1*Δ*psc1*Δ uninduced (without galactose). We required (i) ≥20 total depth and (ii) ≥20 G reads in each of the biological replicates to be considered a true editing event. Applying these criteria yielded 95 high-confidence A–G edited transcripts. Of the 95 transcripts, deletion strains for 74 were available in the partial genome deletion collection and were screened for CO_2_ sensitivity.

### Competition assay

The competition assay was performed as previously reported in reference 7. Using a 96-well plate, the H99 wild-type mNeonGreen-labeled reference strain and individual unlabeled deletion strains were incubated overnight at 30°C in 200 μL of YPD. Overnight cultures were diluted and inoculated at a 1:1 ratio (H99 wild-type mNeonGreen: unlabeled deletion strain), at 2 × 10^4^ cells/mL final concentration. The cocultures were grown for 24 h in YPD at ambient conditions or 5% CO_2_. The cocultures were analyzed on the Attune NxT Flow Cytometer with CytKick autosampler and Attune Cytometric software. A histogram plot with 100,000 cells per sample was used to identify mNG-negative and -positive populations. To determine the competitive fitness in CO_2_ relative to ambient conditions, the negative mNeonGreen percentage of cells in CO_2_ was divided by the negative mNeonGreen percentage of cells in ambient conditions. A chi-squared test was performed in Microsoft Excel to determine statistical significance. For unlabeled deletion strains that were deficient in ambient conditions, a two-sided Student’s *t*-test was used to compare ambient to high CO_2_ competitive fitness.

### Quantification and statistical analysis

All statistical analyses reported in the figure legends and text were performed using GraphPad Prism software. The statistical tests employed and sample sizes are indicated in the figure legends. A *P*-value less than 0.05 was considered statistically significant and *P < 0.05; **P < 0.01; ***P < 0.001; and ****P < 0.0001.

## Data Availability

The RNA sequencing data generated in this publication have been deposited in the NCBI Sequence Read Archive (SRA), accession number NCBI BioProject: PRJNA1331806. RNA sequencing data were analyzed using the *C. neoformans* H99 reference genome (FungiDB version Release 59) from the FungiDB database. Materials generated in this study will be made available upon request, and the source data are provided with this paper. This paper does not report original code. All unique/stable reagents generated in this study are available from the corresponding author with a completed materials transfer agreement.

## SUPPLEMENTAL MATERIAL

Supplemental material mbio.02051-26-s0001.pdf Figures 1–4 and Tables 1–4.

ASM does not own the copyrights to Supplemental Material that may be linked to, or accessed through, an article. The authors have granted ASM a non-exclusive, world-wide license to publish the Supplemental Material files. Please contact the corresponding author directly for reuse.

## References

[1] Denning DW. 2024. Global incidence and mortality of severe fungal disease. Lancet Infect Dis 24:e428–e438. doi:10.1016/S1473-3099(23)00692-8 38224705

[2] Krysan DJ, Zhai B, Beattie SR, Misel KM, Wellington M, Lin X. 2019. Host carbon dioxide concentration is an independent stress for *Cryptococcus neoformans* that affects virulence and antifungal susceptibility. mBio 10:01410–01419. doi:10.1128/mBio.01410-19 PMC6606813 31266878

[3] Bahn Y-S, Cox GM, Perfect JR, Heitman J. 2005. Carbonic anhydrase and CO_2_ sensing during *Cryptococcus neoformans* growth, differentiation, and virulence. Curr Biol 15:2013–2020. doi:10.1016/j.cub.2005.09.047 16303560

[4] Blackburn EE, Ristow LC, Lin X, Krysan DJ. 2025. Host-induced climate change: carbon dioxide tolerance as a *Cryptococcus neoformans* virulence trait. PLoS Pathog 21:e1013351. doi:10.1371/journal.ppat.1013351 PMC12333976 40779504

[5] Chadwick BJ, Lin X. 2024. Effects of CO_2_ in fungi. Curr Opin Microbiol 79:102488. doi:10.1016/j.mib.2024.102488 PMC11162916 38759247

[6] Chadwick BJ, Pham T, Xie X, Ristow LC, Krysan DJ, Lin X. 2022. The RAM signaling pathway links morphology, thermotolerance, and CO_2_ tolerance in the global fungal pathogen *Cryptococcus neoformans*. eLife 11:e82563. doi:10.7554/eLife.82563 PMC9708076 36416414

[7] Ristow LC, Jezewski AJ, Chadwick BJ, Stamnes MA, Lin X, Krysan DJ. 2023. *Cryptococcus neoformans* adapts to the host environment through TOR-mediated remodeling of phospholipid asymmetry. Nat Commun 14:6587. doi:10.1038/s41467-023-42318-y PMC10584969 37852972

[8] Hergovich A, Stegert MR, Schmitz D, Hemmings BA. 2006. NDR kinases regulate essential cell processes from yeast to humans. Nat Rev Mol Cell Biol 7:253–264. doi:10.1038/nrm1891 16607288

[9] Udan RS, Kango-Singh M, Nolo R, Tao C, Halder G. 2003. Hippo promotes proliferation arrest and apoptosis in the Salvador/Warts pathway. Nat Cell Biol 5:914–920. doi:10.1038/ncb1050 14502294

[10] Justice RW, Zilian O, Woods DF, Noll M, Bryant PJ. 1995. The *Drosophila* tumor suppressor gene warts encodes a homolog of human myotonic dystrophy kinase and is required for the control of cell shape and proliferation. Genes Dev 9:534–546. doi:10.1101/gad.9.5.534 7698644

[11] Nelson B, Kurischko C, Horecka J, Mody M, Nair P, Pratt L, Zougman A, McBroom LDB, Hughes TR, Boone C, Luca FC. 2003. RAM: a conserved signaling network that regulates Ace2p transcriptional activity and polarized morphogenesis. Mol Biol Cell 14:3782–3803. doi:10.1091/mbc.e03-01-0018 PMC196567 12972564

[12] Bidlingmaier S, Weiss EL, Seidel C, Drubin DG, Snyder M. 2001. The Cbk1p pathway is important for polarized cell growth and cell separation in *Saccharomyces cerevisiae*. Mol Cell Biol 21:2449–2462. doi:10.1128/MCB.21.7.2449-2462.2001 PMC86877 11259593

[13] Colman-Lerner A, Chin TE, Brent R. 2001. Yeast Cbk1 and Mob2 activate daughter-specific genetic programs to induce asymmetric cell fates. Cell 107:739–750. doi:10.1016/s0092-8674(01)00596-7 11747810

[14] Weiss EL, Kurischko C, Zhang C, Shokat K, Drubin DG, Luca FC. 2002. The *Saccharomyces cerevisiae* Mob2p-Cbk1p kinase complex promotes polarized growth and acts with the mitotic exit network to facilitate daughter cell-specific localization of Ace2p transcription factor. J Cell Biol 158:885–900. doi:10.1083/jcb.200203094 PMC2173146 12196508

[15] Yang J, McCormick MA, Zheng J, Xie Z, Tsuchiya M, Tsuchiyama S, El-Samad H, Ouyang Q, Kaeberlein M, Kennedy BK, Li H. 2015. Systematic analysis of asymmetric partitioning of yeast proteome between mother and daughter cells reveals “aging factors” and mechanism of lifespan asymmetry. Proc Natl Acad Sci USA 112:11977–11982. doi:10.1073/pnas.1506054112 PMC4586869 26351681

[16] Higuchi-Sanabria R, Pernice WMA, Vevea JD, Alessi Wolken DM, Boldogh IR, Pon LA. 2014. Role of asymmetric cell division in lifespan control in *Saccharomyces cerevisiae*. FEMS Yeast Res 14:1133–1146. doi:10.1111/1567-1364.12216 PMC4270926 25263578

[17] Henderson KA, Hughes AL, Gottschling DE. 2014. Mother-daughter asymmetry of pH underlies aging and rejuvenation in yeast. eLife 3:e03504. doi:10.7554/eLife.03504 PMC4175738 25190112

[18] Walton FJ, Heitman J, Idnurm A. 2006. Conserved elements of the RAM signaling pathway establish cell polarity in the basidiomycete *Cryptococcus neoformans* in a divergent fashion from other fungi. Mol Biol Cell 17:3768–3780. doi:10.1091/mbc.e06-02-0125 PMC1556378 16775005

[19] Wakade RS, Ristow LC, Stamnes MA, Kumar A, Krysan DJ. 2020. The NDR/LATS kinase Cbk1 regulates a specific subset of Ace2 functions and suppresses the hypha-to-yeast transition in *Candida albicans*. mBio 11:01900–01920. doi:10.1128/mBio.01900-20 PMC7439482 32817109

[20] Gates KL, Howell HA, Nair A, Vohwinkel CU, Welch LC, Beitel GJ, Hauser AR, Sznajder JI, Sporn PHS. 2013. Hypercapnia impairs lung neutrophil function and increases mortality in murine *Pseudomonas* pneumonia. Am J Respir Cell Mol Biol 49:821–828. doi:10.1165/rcmb.2012-0487OC PMC3931098 23777386

[21] Mazanka E, Alexander J, Yeh BJ, Charoenpong P, Lowery DM, Yaffe M, Weiss EL. 2008. The NDR/LATS family kinase Cbk1 directly controls transcriptional asymmetry. PLoS Biol 6:e203. doi:10.1371/journal.pbio.0060203 PMC2517623 18715118

[22] Gógl G, Schneider KD, Yeh BJ, Alam N, Nguyen Ba AN, Moses AM, Hetényi C, Reményi A, Weiss EL. 2015. The structure of an NDR/LATS kinase-Mob complex reveals a novel kinase-coactivator system and substrate docking mechanism. PLoS Biol 13:e1002146. doi:10.1371/journal.pbio.1002146 PMC4428629 25966461

[23] Piao W, Li C, Sun P, Yang M, Ding Y, Song W, Jia Y, Yu L, Lu Y, Jin H. 2023. Identification of RNA-binding protein targets with hyperTRIBE in *Saccharomyces cerevisiae* Int J Mol Sci 24:9033. doi:10.3390/ijms24109033 PMC10218906 37240377

[24] McMahon AC, Rahman R, Jin H, Shen JL, Fieldsend A, Luo W, Rosbash M. 2016. TRIBE: hijacking an RNA-editing enzyme to identify cell-specific targets of RNA-binding proteins. Cell 165:742–753. doi:10.1016/j.cell.2016.03.007 PMC5027142 27040499

[25] Sankaranarayanan S, Haag C, Petzsch P, Köhrer K, Matuszyńska A, Zarnack K, Feldbrügge M. 2023. The mRNA stability factor Khd4 defines a specific mRNA regulon for membrane trafficking in the pathogen *Ustilago maydis* Proc Natl Acad Sci USA 120:e2301731120. doi:10.1073/pnas.2301731120 PMC10450656 37590419

[26] Ristow LC, Blackburn EE, Jezewski AJ, Lin X, Krysan DJ. 2026. *Cryptococcus neoformans* adapts to host CO_2_ concentrations via metabolic and stress-response remodeling. PLoS Biol 24:e3003561. doi:10.1371/journal.pbio.3003561 PMC13160432 42085498

[27] Boucher MJ, Banerjee S, Joshi MB, Wei AL, Nalley MJ, Huang MY, Lei S, Ciranni M, Condon A, Langen A, Goddard TD, Caradonna I, Goranov AI, Homer CM, Mortensen Y, Petnic S, Reilly MC, Xiong Y, Susa KJ, Pastore VP, Zaro BW, Madhani HD. 2025. Phenotypic landscape of an invasive fungal pathogen reveals its unique biology. Cell 188:4003–4024. doi:10.1016/j.cell.2025.05.017 PMC12407185 40505656

[28] Shi R, Lin X. 2024. Illuminating the *Cryptococcus neoformans* species complex: unveiling intracellular structures with fluorescent-protein-based markers. Genetics 227:iyae059. doi:10.1093/genetics/iyae059 PMC11228865 38752295

[29] Bi E, Pringle JR. 1996*.* *ZDS1* and *ZDS2*, genes whose products may regulate Cdc42p in *Saccharomyces cerevisiae*. Mol Cell Biol 16:5264–5275. doi:10.1128/MCB.16.10.5264 PMC231526 8816439

[30] Yasutis K, Vignali M, Ryder M, Tameire F, Dighe SA, Fields S, Kozminski KG. 2010. Zds2p regulates Swe1p-dependent polarized cell growth in *Saccharomyces cerevisiae* via a novel Cdc55p interaction domain. Mol Biol Cell 21:4373–4386. doi:10.1091/mbc.E10-04-0326 PMC3002390 20980617

[31] Yakura M, Ozoe F, Ishida H, Nakagawa T, Tanaka K, Matsuda H, Kawamukai M. 2006. *zds1*, a novel gene encoding an ortholog of Zds1 and Zds2, controls sexual differentiation, cell wall integrity and cell morphology in fission yeast. Genetics 172:811–825. doi:10.1534/genetics.105.050906 PMC1456246 16322512

[32] Racki WJ, Bécam AM, Nasr F, Herbert CJ. 2000. Cbk1p, a protein similar to the human myotonic dystrophy kinase, is essential for normal morphogenesis in *Saccharomyces cerevisiae*. EMBO J 19:4524–4532. doi:10.1093/emboj/19.17.4524 PMC302079 10970846

[33] Tutucci E, Vera M, Biswas J, Garcia J, Parker R, Singer RH. 2018. An improved MS2 system for accurate reporting of the mRNA life cycle. Nat Methods 15:81–89. doi:10.1038/nmeth.4502 PMC5843578 29131164

[34] Jansen JM, Wanless AG, Seidel CW, Weiss EL. 2009. Cbk1 regulation of the RNA-binding protein Ssd1 integrates cell fate with translational control. Curr Biol 19:2114–2120. doi:10.1016/j.cub.2009.10.071 PMC2805764 19962308

[35] Kurischko C, Kim HK, Kuravi VK, Pratzka J, Luca FC. 2011. The yeast Cbk1 kinase regulates mRNA localization via the mRNA-binding protein Ssd1. J Cell Biol 192:583–598. doi:10.1083/jcb.201011061 PMC3044126 21339329

[36] Wanless AG, Lin Y, Weiss EL. 2014. Cell morphogenesis proteins are translationally controlled through UTRs by the Ndr/LATS target Ssd1. PLoS One 9:e85212. doi:10.1371/journal.pone.0085212 PMC3897418 24465507

[37] Zheng Y, Bender A, Cerione RA. 1995. Interactions among proteins involved in bud-site selection and bud-site assembly in *Saccharomyces cerevisiae**.* J Biol Chem 270:626–630. doi:10.1074/jbc.270.2.626 7822288

[38] Chant J, Pringle JR. 1995. Patterns of bud-site selection in the yeast *Saccharomyces cerevisiae*. J Cell Biol 129:751–765. doi:10.1083/jcb.129.3.751 PMC2120437 7730409

[39] McBride HJ, Yu Y, Stillman DJ. 1999. Distinct regions of the Swi5 and Ace2 transcription factors are required for specific gene activation. J Biol Chem 274:21029–21036. doi:10.1074/jbc.274.30.21029 10409653

[40] Bertrand E, Chartrand P, Schaefer M, Shenoy SM, Singer RH, Long RM. 1998. Localization of *ASH1* mRNA particles in living yeast. Mol Cell 2:437–445. doi:10.1016/s1097-2765(00)80143-4 9809065

[41] Kelliher CM, Leman AR, Sierra CS, Haase SB. 2016. Investigating conservation of the cell-cycle-regulated transcriptional program in the fungal pathogen, *Cryptococcus neoformans**.* PLoS Genet 12:e1006453. doi:10.1371/journal.pgen.1006453 PMC5137879 27918582

[42] Lin J, Fan Y, Lin X. 2020. Transformation of *Cryptococcus neoformans* by electroporation using a transient CRISPR-Cas9 expression (TRACE) system. Fungal Genet Biol 138:103364. doi:10.1016/j.fgb.2020.103364 PMC7153975 32142753

