# Supplementary material for: The RNA-binding protein Psc1 functions downstream of the NDR/LATS kinase Cbk1 to modulate CO2 tolerance in Cryptococcus neoformans
Source: mBio. 2026 Sep 21;17(10):e02051-26. doi: 10.1128/mbio.02051-26 (PMC13712771; doi:10.1128/mbio.02051-26)
Supplement: Supplementary file 1 [file mbio.02051-26-s0001.pdf]

Supplementary Figures

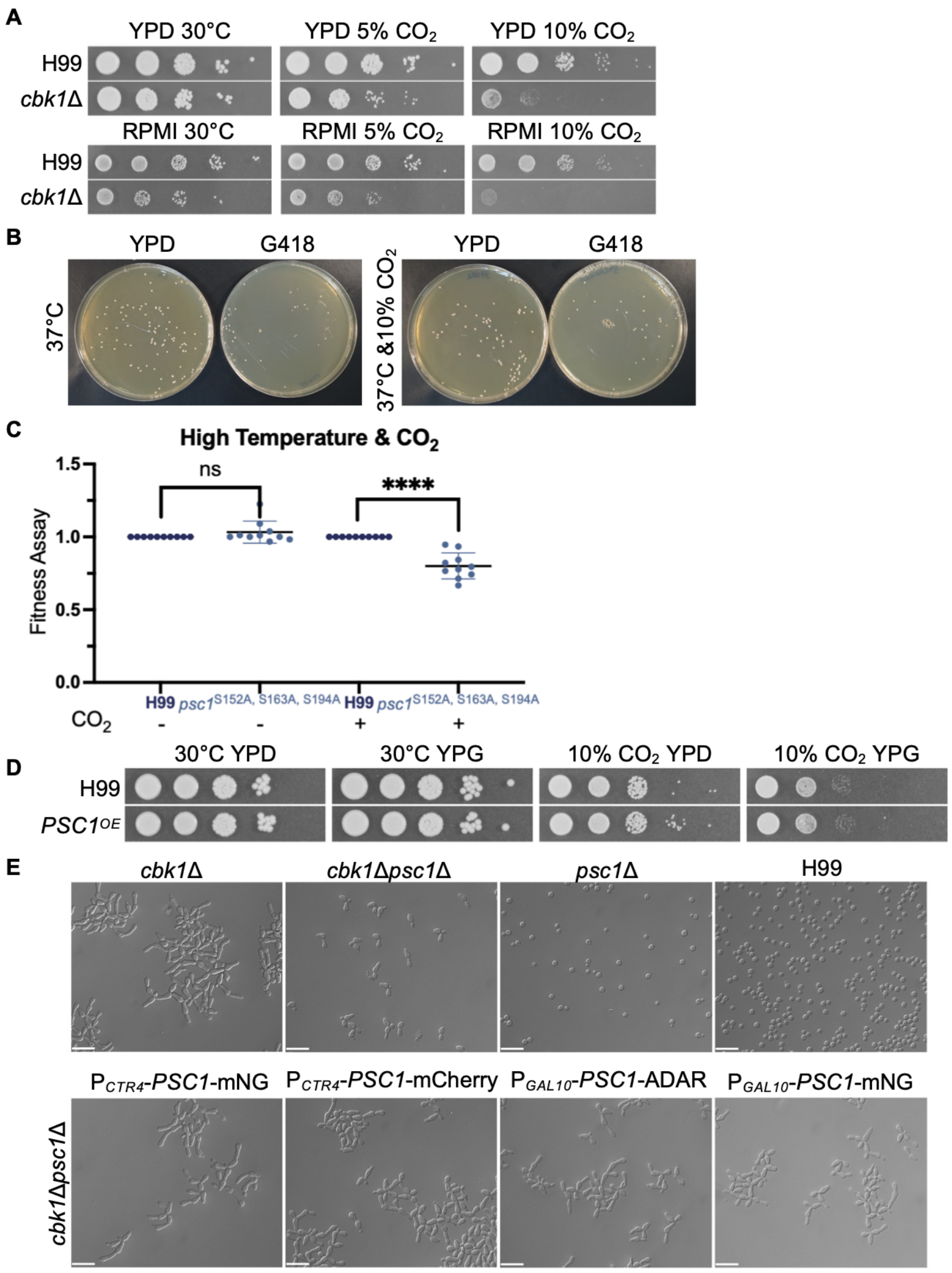

S1 Fig. Overexpression of the wild-type *PSC1* allele has no fitness cost but overexpression of the *psc1* phospho-null allele does have a minor fitness cost. (A) Ten-fold serial dilutions from overnight cultures of wild-type H99 and *cbk1*Δ strains were spotted onto YPD and RPMI media and incubated for 48 hours in ambient CO_2, 5% CO_2, and 10% CO_2 before images were acquired. (B) Representative fitness assay plates used to quantify *psc1* phospho-null allele (*psc1*$^{S152A, S163A, S194A}$) fitness. CFUs on YPD reflect total cell counts and CFUs on YPD+G418 reflect the cell counts of the

mutant alone. (C) Fitness assay of wild-type H99 strain versus a strain expressing the *psc1* phospho-null allele (*psc1*$^{S152A, S163A, S194A}$) at high temperature (37°C) and high CO_2 (10%) quantified by fluorescence (fluorescence *psc1*$^{S152A, S163A, S194A}$-mNG or no fluorescence H99). An unpaired two-tailed *t*-test was performed for both conditions, graph depicts mean ± SEM, line at median (n=10), $P_{37°C\ H99\ vs.\ 37°C\ psc1}{}^{S152A, S163A, S194A}$ = ns (0.1838), $P_{37°C\ \&\ CO2\ H99\ vs.\ 37°C\ \&\ CO2\ psc1}{}^{S152A, S163A, S194A}$ = <0.0001. (D) Ten-fold serial dilutions from overnight cultures of wild-type H99 and *PSC1*^{OE} strains were spotted onto YPD (non-inducing) and YPG (inducing) media and incubated for 72 hours in ambient CO_2 or high CO_2 before images were acquired. (E) Images were captured after 16 hours of induction on galactose (*GAL10* promoter) or YPD with BCS (*CTR4* promoter); the scale bar indicates 20 μm.

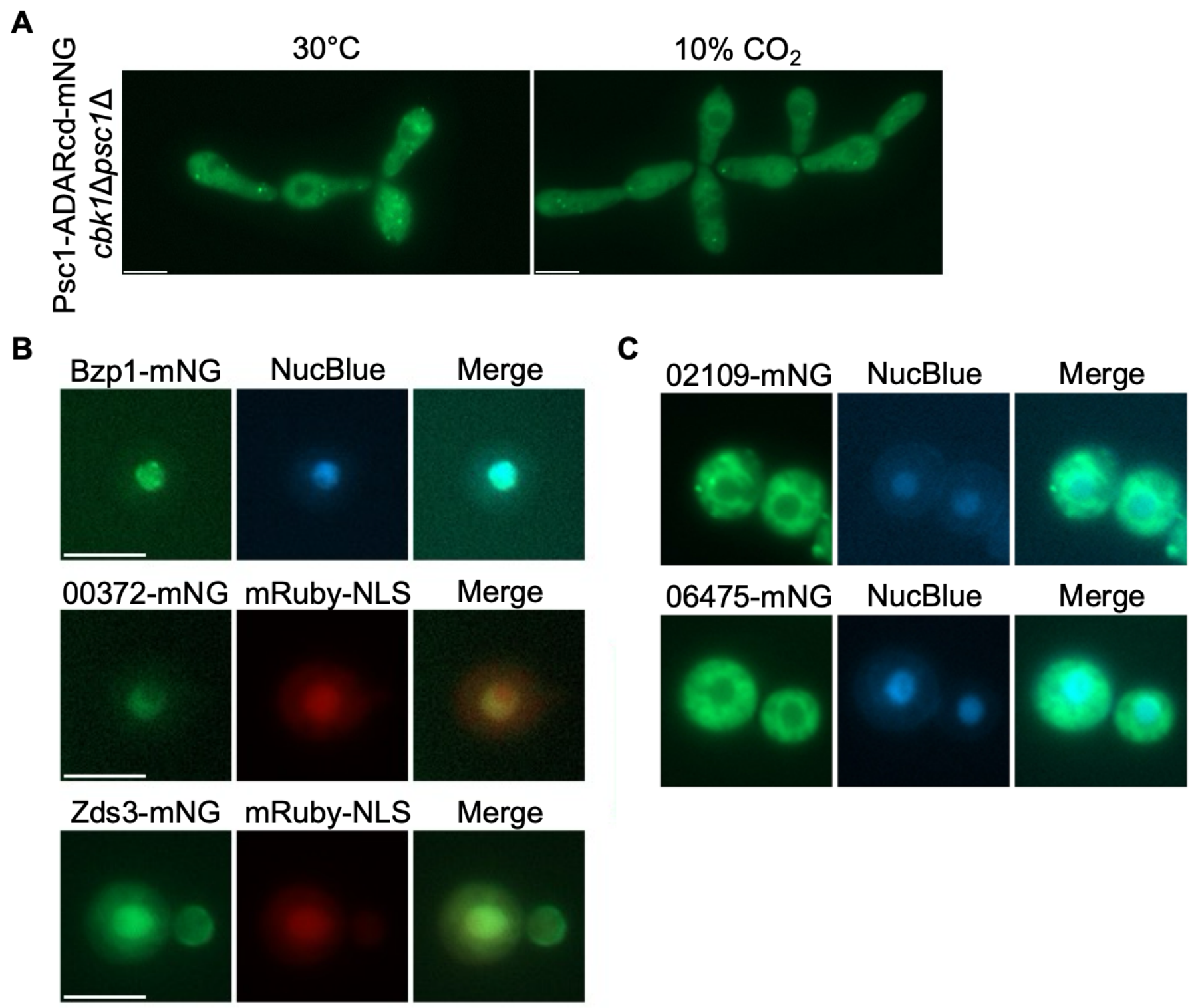

S2 Fig. Addition of ADARcd does not disrupt Psc1 condensate formation.

(A, B, and C) The scale bar indicates 5 µm. (A) Images were captured after six hours of induction with BCS (100 µM); the first four hours at 30°C, followed by a two-hour shift to 10% CO_2 for the high CO_2 condition. For ambient CO_2, the cells remained at 30°C for two additional hours. (B) Cells expressing Bzp1 were stained with NucBlue Live Ready Probes Reagent, which stains nuclear DNA. Cells expressing CNAG_00372 and Zds3 were crossed with JEC20 *MAT*a expressing an mRuby-NLS reporter and visualized. (C) Cells expressing CNAG_02109 and CNAG_06475 were stained with NucBlue Live Ready Probes Reagent to visualize nuclei and perinuclear enrichment was observed.

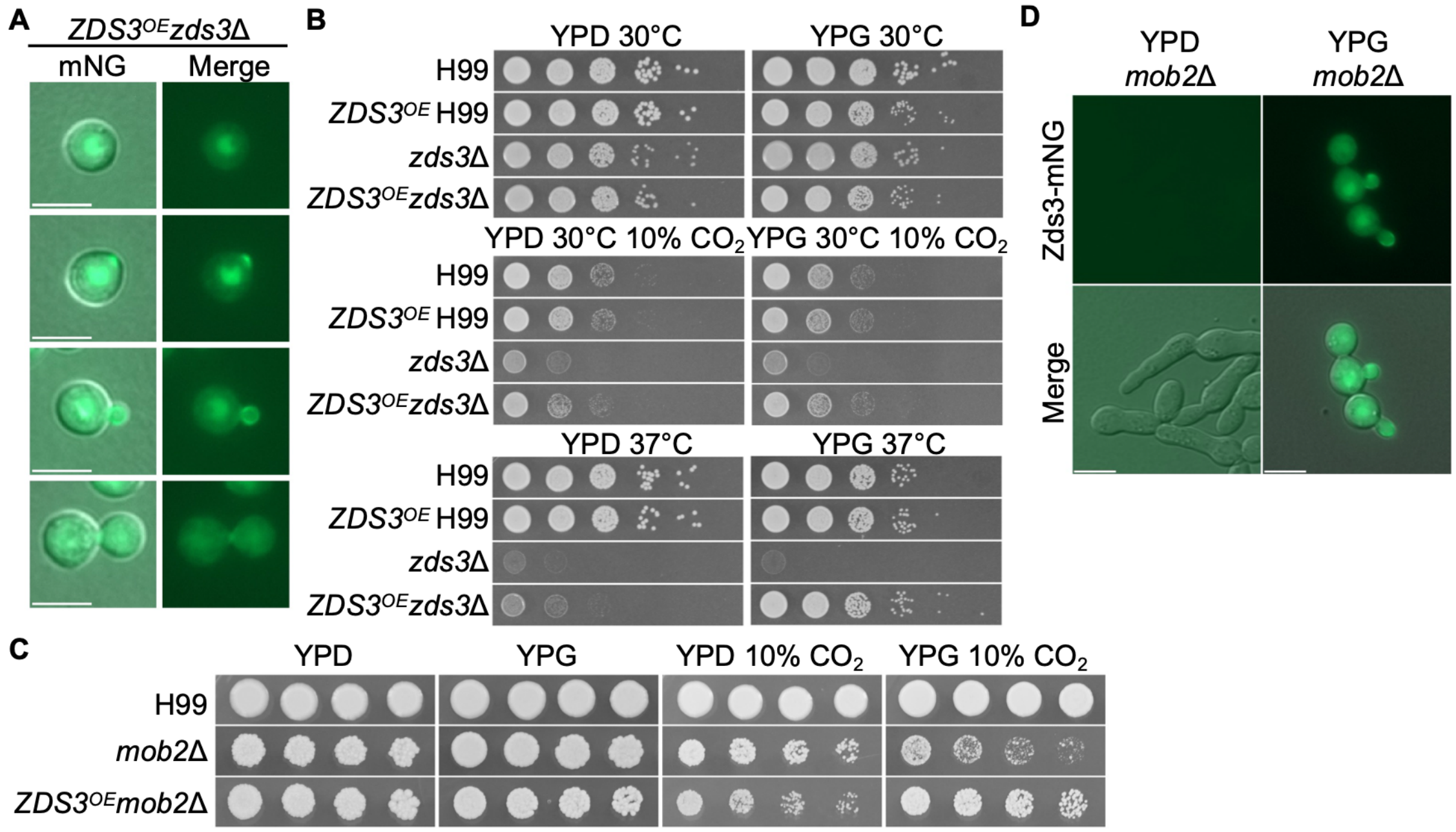

S3 Fig. Overexpression of *ZDS3* rescues the *zds3*Δ mutant phenotypes and partially restores the *mob2*Δ mutant defects.

(A) Subcellular localization pattern of overexpressed Zds3-mNG in the *zds3*Δ mutant; the scale bar indicates 5 µm. Images were acquired after six hours of induction with galactose. Overexpression of *ZDS3* confers no changes to cell morphology. (B) Ten-fold serial dilutions from overnight cultures of wild-type H99, *ZDS3*^{OE}, *zds3*Δ, and *ZDS3*^{OE}*zds3*Δ were spotted onto YPD (non-inducing) and YPG (inducing) media and incubated for 48 hours at 30°C, 37°C, and 30°C with 10% CO_2 before images were acquired. (C) 2.5-fold serial dilutions from overnight cultures of wild-type H99, *mob2*Δ, and *ZDS3*^{OE}*mob2*Δ strains were spotted onto YPD (non-inducing) and YPG (inducing) media and incubated for 72 hours in ambient CO_2 or high CO_2 before images were acquired. (D) Images were taken after eight hours of induction with galactose or eight hours of non-inducing conditions, both at 30°C; the scale bar indicates 5 µm.

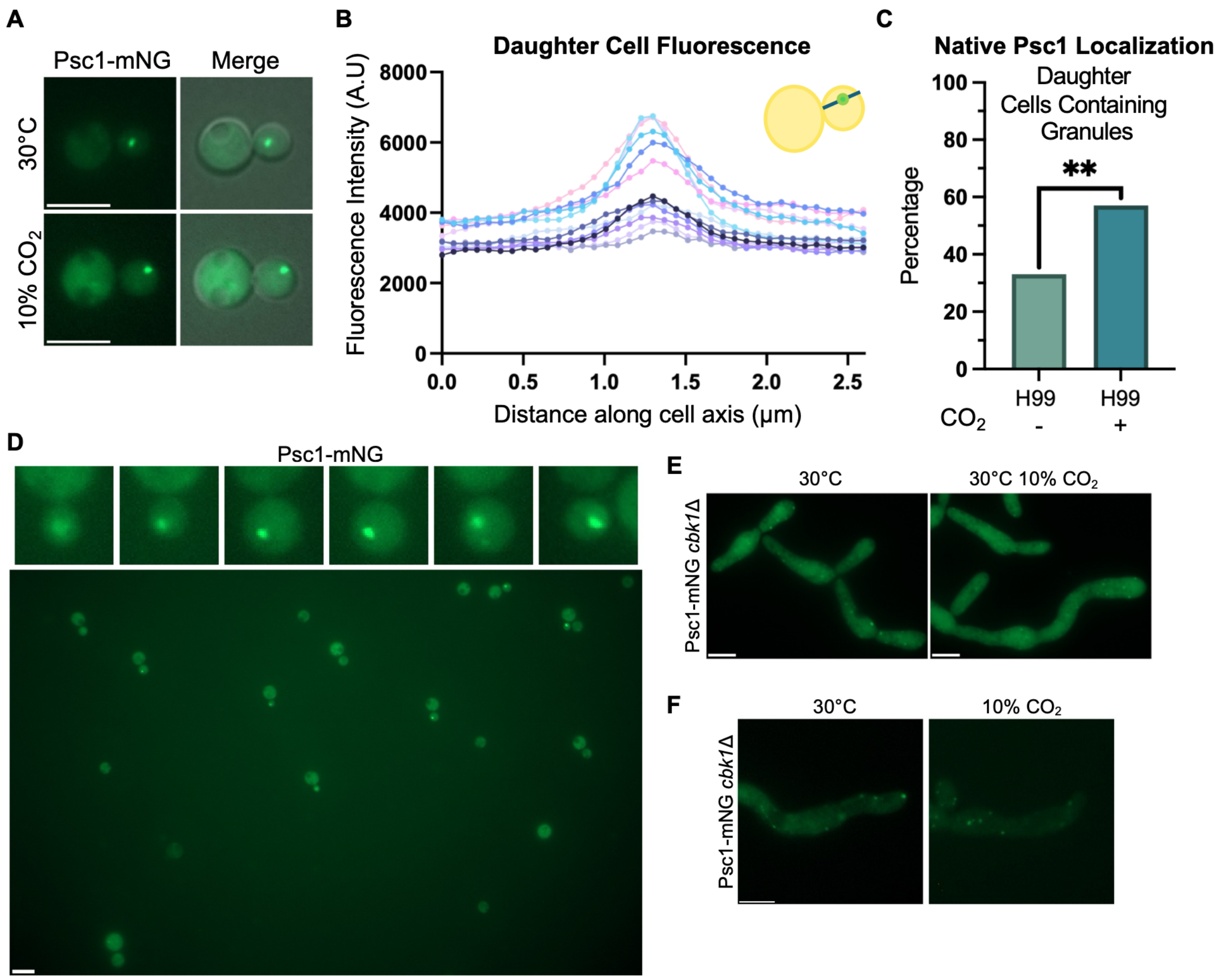

S4 Fig. Psc1 expressed by its native promoter localizes asymmetrically to daughter cells in response to high CO_2.

(A, D, E, and F) Images were captured after six hours of growth on YPD; the first four hours at 30°C, followed by a two-hour shift to 10% CO_2. For ambient CO_2, the cells remained at 30°C for two additional hours. The scale bar indicates 5 µm. (A) Psc1 localization in the wild-type strain in ambient CO_2 and 10% CO_2. (B) Using ImageJ, a line (3.0 µm) was drawn through daughter cells expressing Psc1 driven by its native promoter. This is to measure the fluorescence intensity across the daughter cell with a Psc1 condensate (n=13 daughter cells, each with one Psc1 foci). The lines were drawn

bisecting the Psc1 foci. Fluorescence intensity and distance along with cell axis were centered at the maximum fluorescence signal. (C) At least five independent experimental replicates were used for quantification. A two-sided Fisher's exact test was performed. At least 61 budding cells were quantified for each condition. $n_{30°C}$ = 62 and $n_{10\%CO2}$ = 61; $P_{30°C\ vs.\ 10\%\ CO2}$ = 0.0077. (D) Psc1 granules are similar in shape with variable size in the daughter cells. (E) Extended image of Psc1 localization in the *cbk1*Δ mutant in ambient CO_2 and high CO_2. (F) Localization of Psc1 driven by its native promoter in the *cbk1*Δ mutant in ambient CO_2 and 10% CO_2.

Supplementary Tables

S1 Table. 95 HyperTRIBE targets of Psc1.

CNAG_00079
CNAG_00116
CNAG_00149
CNAG_00150
CNAG_00157
CNAG_00250
CNAG_00254
CNAG_00261
CNAG_00371
CNAG_00372
CNAG_00373
CNAG_00457
CNAG_00730
CNAG_00752
CNAG_00790
CNAG_00799
CNAG_01004
CNAG_01024
CNAG_01097
CNAG_01174
CNAG_01223
CNAG_01258
CNAG_01272
CNAG_01421
CNAG_01441
CNAG_01533
CNAG_01562
CNAG_01657
CNAG_01739
CNAG_01743
CNAG_01778
CNAG_01796
CNAG_01813
CNAG_01890
CNAG_01895

CNAG_01985
CNAG_02008
CNAG_02109
CNAG_02144
CNAG_02214
CNAG_02296
CNAG_02300
CNAG_02445
CNAG_02510
CNAG_02585
CNAG_02665
CNAG_02814
CNAG_02816
CNAG_02822
CNAG_03385
CNAG_03397
CNAG_03482
CNAG_03856
CNAG_03857
CNAG_04183
CNAG_04197
CNAG_04245
CNAG_04290
CNAG_04437
CNAG_04633
CNAG_04634
CNAG_04687
CNAG_04821
CNAG_04861
CNAG_04867
CNAG_04876
CNAG_04952
CNAG_04963
CNAG_04991
CNAG_05080
CNAG_05124
CNAG_05247
CNAG_05264
CNAG_05372

CNAG_05398
CNAG_05724
CNAG_05791
CNAG_05807
CNAG_05893
CNAG_05918
CNAG_06109
CNAG_06134
CNAG_06201
CNAG_06320
CNAG_06344
CNAG_06396
CNAG_06422
CNAG_06475
CNAG_06623
CNAG_06782
CNAG_06795
CNAG_06920
CNAG_06998
CNAG_07108
CNAG_07177

S2 Table. Strain list. Cryptococcus neoformans strains used in this study. Identifiers of all C. neoformans strains used in this study.

***C. neoformans* strain Identifier**	**Strain Background**	**Genotype**	**Reference**	**Additional information**
H99	H99	*MAT*α	[1]	Original WT
*cbk1*Δ [cbk1::NAT]	H99	*MAT*α	[2]	BC1024
*cbk1*Δ*psc1*Δ [cbk1::NAT, psc1::HYG]	H99	*MAT*α	[2]	BC1369
psc*1*Δ [psc1::HYG]	H99	*MAT*α	[2]	BC1393
P_{CTR4}-*PSC1*-mNeonGreen-G418	H99	*MAT*α	Here	EEB173
P_{GAL10}-*PSC1*-mNeonGreen-G418	H99	*MAT*α	Here	EEB428
P_{CTR4}-*PSC1*-mCherry-G418	H99	*MAT*α	Here	EEB183
P_{GAL10}-*PSC1* S152A, S163A, S194A-mNeonGreen-G418	H99	*MAT*α	Here	EEB575
P_{CTR4}-*PSC1*-mNeonGreen-G418	*cbk1*Δ	*MAT*α	Here	EEB420
P_{GAL10}-*PSC1*-mNeonGreen-G418	*cbk1*Δ	*MAT*α	Here	EEB439-442
P_{CTR4}-*PSC1*-mNeonGreen-G418	*cbk1*Δ*psc1*Δ	*MAT*α	Here	EEB177
P_{GAL10}-*PSC1*-mNeonGreen-G418	*cbk1*Δ*psc1*Δ	*MAT*α	Here	EEB436
P_{CTR4}-*PSC1*-mCherry-G418	*cbk1*Δ*psc1*Δ	*MAT*α	Here	EEB424
P_{GAL10}-*PSC1*-ADAR-G418	*cbk1*Δ*psc1*Δ	*MAT*α	Here	EEB153-155

P_{GAL10}-*ZDS3*-mNeonGreen-G418	H99	*MAT*α	Here	EEB276
P_{GAL10}-*CNAG_04290*-mNeonGreen-G418	H99	*MAT*α	Here	EEB237
P_{GAL10}-*BZP1*-mNeonGreen-G418	H99	*MAT*α	Here	EEB240
P_{GAL10}-*CNAG_01258*-mNeonGreen-G418	H99	*MAT*α	Here	EEB284
P_{GAL10}-*CNAG_00372*-mNeonGreen-G418	H99	*MAT*α	Here	EEB243
P_{GAL10}-*CNAG_01024*-mNeonGreen-G418	H99	*MAT*α	Here	EEB279
P_{GAL10}-*CNAG_02109*-mNeonGreen-G418	H99	*MAT*α	Here	EEB306
P_{GAL10}-*CNAG_06475*-mNeonGreen-G418	H99	*MAT*α	Here	EEB248
P_{GAL10}-*ZDS3*-mNeonGreen-G418	*zds3*Δ	*MAT*α	Here	EEB377
P_{GAL10}-*ZDS3*-mNeonGreen-G418	*cbk1*Δ	*MAT*α	Here	EEB327
*zds3*Δ	H99	*MAT*α	[3]	Madhani collection, plate 36, well G04
P_{GAL10}-*ZDS3*-mNeonGreen-G418	*cna1*Δ	*MAT*α	Here	EEB370
P_{GAL10}-*ZDS3*-12 MS2 binding sites-NAT	H99	MATα	Here	EEB397
P_{GPD1}-MCP-mNeonGreen-HYG	H99	*MAT*α	Here	EEB387
NAT-P_{GAL10}-5’UTR-*ZDS3*-12 MS2 binding sites -3’UTR-T_{GPD1}	P_{GPD1}-MCP-mNeonGreen-HYG	*MAT*α	Here	EEB401

NAT-P_{GAL10}-5'UTR-*ZDS3*-12 MS2 binding sites -3'UTR-T_{GPD1}	P_{GPD1}-MCP-mNeonGreen-HYG & P_{CTR4}-*PSC1*-mCherry-G418	*MAT*α	Here	EEB404
NAT-P_{GAL10}-5'UTR-*ZDS3*-12 MS2 binding sites -3'UTR-T_{GPD1} [*cbk1*Δ]	P_{GPD1}-MCP-mNeonGreen-HYG & P_{CTR4}-*PSC1*-mCherry-G418	*MAT*α	Here	EEB409
*cna1*Δ::NAT	H99	*MAT*α	Here	YF281
P_{GAL10}-*ZDS3*-mNeonGreen-G418	*mob2*Δ	*MAT*α	Here	EEB551
*mob2*Δ	H99	*MAT*α	[4]	FJW10
Native Promoter-*PSC1*-mNeonGreen-G418	H99	*MAT*α	Here	EEB555
Native Promoter-*PSC1*-mNeonGreen-G418	*cbk1*Δ	*MAT*α	Here	EEB559
P_{CTR4}-*PSC1*-ADAR-mNG-G418	*cbk1*Δ*psc1*Δ	*MAT*α	Here	EEB86
P_{GAL10}-*ZDS3*-mNeonGreen-G418	P_{TEF1}-mRuby3-NLS-HYG		Here	EEB419
P_{GAL10}-*CNAG_00372*-mNeonGreen-G418	P_{TEF1}-mRuby3-NLS-HYG		Here	EEB618
P_{TEF1}-mRuby3-NLS-HYG	JEC20	*MATa*	[5]	RSCR234

S3 Table. Recombinant DNA. Summary of all recombinant DNA plasmids and constructs used in this study.

Recombinant DNA	Source	Identifier
P_{CTR4}-*PSC1*-mNeonGreen-G418	Here	2664
P_{GAL10}-*PSC1*-mNeonGreen-G418	Here	2990
P_{CTR4}-*PSC1*-mCherry-G418	Here	2793
P_{GAL10}-*PSC1*[S to A for S152, 163, 194]-mNeonGreen-G418	Here	3125
P_{GAL10}-*PSC1*-ADARcd-G418	Here	2725
P_{GAL10}-*ZDS3*-mNeonGreen-G418	Here	2892
P_{GAL10}-*CNAG_04290*-mNeonGreen-G418	Here	2884
P_{GAL10}-*BZP1*-mNeonGreen-G418	Here	2885
P_{GAL10}-*CNAG_01258*-mNeonGreen-G418	Here	2894

P_{GAL10}-*CNAG_00372*-mNeonGreen-G418	Here	2886
P_{GAL10}-*CNAG_01024*-mNeonGreen-G418	Here	2893
P_{GAL10}-*CNAG_02109*-mNeonGreen-G418	Here	2896
P_{GAL10}-*CNAG_06475*-mNeonGreen-G418	Here	2887
P_{GPD1}-MCP-mNeonGreen-HYG	Here	2943
pUC57 - MCP	Here	2937
pUC57 - partial *ZDS3* ORF - 12 MS2 binding sites - Zds3 3' UTR	Here	2940
NAT-P_{GAL10}-5'UTR-*ZDS3*-12 MS2 binding sites -3'UTR-T_{GPD1}		Linear Construct Fusion Assembled
P_{CRG1}-*KHD4*-3xGS-ADARcd-eGFP-nosT-AmpR	Gift from Dr. Michael Feldbrügge	pUMa4057

Native Promoter-*PSC1*-mNeonGreen-G418	Here	3115
P_{CTR4}-*PSC1*-ADARcd-mNeonGreen-G418	Here	2528

S4 Table. Primers. Primers used for strain and construct generation. Primer sequence and use description for all primers used to generate *C. neoformans* strains and recombinant DNA products.

Primer	Use/Description	Sequence
LinLab8639	Delete *CBK1*gRNA-U6 reverse	ggtacgtcgaagacggagatAACAGTATACCCTGCCGGTG
LinLab8640	Delete *CBK1*gRNA-scaffold forward	atctccgtcttcgacgtaccGTTTTAGAGCTAGAAATAGCAAGTT
LinLab4627	gRNA-U6 forward	ggctcaaagagcagatcaatg
LinLab4722	SH2 gRNA-U6 reverse	agactccacagcctaagatcAACAGTATACCCTGCCGGTG
LinLab4628	gRNA-scaffold reverse	cctctgacacatgcagctcc
LinLab4723	SH2 gRNA-scaffold forward	gatcttaggctgtggagtctGTTTTAGAGCTAGAAATAGCAAGTT
LinLab4594	gRNA forward	ccatcgatttgcattagaactaaaaacaaagca
LinLab4595	gRNA reverse	ccgctcgagtaaaacaaaaaagcaccgac
M13F	Amplification of all overexpression constructs forward	GTAAAACGACGGCCAG
M13R	Amplification of all overexpression constructs reverse	CAGGAAACAGCTATGAC
LinLab11016/EEB	*PSC1* ORF Restriction Enzyme ApaI for cloning p2990 forward	gggcccATGTCCATCTCCCTCCCC
LinLab11017/EEB	*PSC1* ORF Restriction Enzyme FseI for cloning p2990 reverse	gggccggccTCCTTGCGTCTCGACACT

LinLab9714/EEB	ADAR Restriction Enzyme FseI forward	GGAGGCCGGCCAGGAGGCGGTGGTTCC
LinLab10121/EEB	ADAR Restriction Enzyme PacI reverse	aTTAATTAATTAGGATTCTGCCAGTCCGAACTC
LinLab10718/EEB	*ZDS3* ORF forward	TACCATGgggcccATGTCAAACCAAGTAT
LinLab10719/EEB	*ZDS3* ORF reverse	caccatgggccggccTCGGTGATGGCCATAT
LinLab10635/EEB	*BZP1* ORF forward	agggcccATGGCTACCGCTGTCGCTTCT
LinLab10636/EEB	*BZP1* ORF reverse	ggccggccTCGCCTCATCCCAACTCTCGA
LinLab10633/EEB	*CNAG_04290* ORF forward	agggcccATGCCAGGTCCCCTCTACCGT
LinLab10634/EEB	*CNAG_04290* ORF reverse	ggccggccCATCGCAAGTGTCTTTGGGTG
LinLab10759/EEB	*CNAG_01258* ORF forward	gggcccATGCTGTATCGCCCA
LinLab10760/EEB	*CNAG_01258* ORF reverse	gggccggccCCGCCGAGCAGAGTT
LinLab10693/EEB	*CNAG_00372* ORF forward	gggcccATGGGCGGCATTAGC
LinLab10694/EEB	*CNAG_00372* ORF reverse	gggccggccTATTTTTTTTGCACG
LinLab10720/EEB	*CNAG_01024* ORF forward	GgggcccATGGCGCACGCACCCC
LinLab10721/EEB	*CNAG_01024* ORF reverse	tgggccggccTTGAACGGTGTCTGG
LinLab10722/EEB	*CNAG_02109* ORF forward	GTTACCATGgggcccATGGCATCTCCCCTG
LinLab10723/EEB	*CNAG_02109* ORF reverse	caccatgggccggccTTCAATACCGTCAAA

LinLab10637/EEB	*CNAG_06475* ORF forward	agggcccATGGCCGACCAGCTCGCCACC
LinLab10638/EEB	*CNAG_06475* ORF reverse	ggccggccCTGCTGCCAGTGAGAATAGGT
LinLab10899/EEB	MCP amplified from p2937 forward	ggccggccATGGCTTCTAACTTTA
LinLab10900/EEB	MCP amplified from p2937 homology to mNG reverse	gcccttggacaccatGTAGATGCCGGAGTT
LinLab10901/EEB	mNG homology to MCP forward	CATCTACatggtgtccaagggc
LinLab10902/EEB	mNG reverse	ttaattaattacttgtacagctcgtcc
LinLab10903/EEB	*GAL10* promoter for MS2 forward	ctgGCGGGGAGTACAGGC
LinLab10904/EEB	*GAL10* homology to 5' UTR *ZDS3* for MS2 reverse	ggcatacagttgtaACATGGTAACTCGAGT
LinLab10905/EEB	5' UTR *ZDS3* for MS2 forward	atgTtacaactgtatgcctcc
LinLab10914/EEB	*ZDS3* middle of ORF for MS2 reverse	GGCTGTCCTTGACTCTGT
LinLab10913/EEB	*ZDS3* middle of ORF (MS2 amplify stem loops and UTR), amplified from p2940 forward	GAGTCAAGGACAGCCGCAGGGATACTATGGTGG
LinLab10908/EEB	3'UTR (from GenScript plasmid with MS2 stem loops), amplified from p2940 reverse	ttaattaaaatagtggagtactgtt
Linlab4025/YF	NAT forward	GCTGGCACGACAGGTTTCC
LinLab10897/EEB	NAT homology to *GAL10* reverse	GCTTAGCCTGTACTCCCCGCcagtgtgatggatat

LinLab10898/EEB	*GPD1* terminator homology to UTR forward	tactccactattttaattaagggttacagtag
Linlab3488/YZ	*GPD1* terminator reverse	*GPD1* terminator reverse
LinLab11018/EEB	*PSC1* S194A reverse	AGAGCCGGCGCCTGATCTGTGGTG
LinLab11019/EEB	*PSC1* S194A forward	TCAGGCGCCGGCTCTTCTTCCTGG
LinLab11319/EEB	*PSC1* S152A, S163A reverse	ACCAGAGATggcGCCTGTTTTTGAGTGGTGGATGTTGGTCACGGCCATTGCCCGGCTATG
LinLab11320/EEB	*PSC1* S152A, S163A forward	GGGCAATGGCCGTGACCAACATCCACCACTCAAAAACAGGCgccATCTCTGGTGCACTT
LinLab11317/EEB	*PSC1* Native Promoter forward	acccggggcggccgcAGACGAAGGACAAGA
LinLab11318/EEB	*PSC1* Native Promoter reverse	GATGGACATgggcccGGCTGATGTGGATTT
LinLab8380/BC	*PSC1* gRNA-U6 reverse	tagttgttttcgccgacgccAACAGTATACCCTGCCGGTG
LinLab8381/BC	*PSC1* gRNA-scaffold forward	ggcgtcggcgaaaacaactaGTTTTAGAGCTAGAAATAGCAAGTT
LinLab11327/EEB	Delete *MOB2* gRNA-U6 reverse	ACCAACACTCCCACTCAGCAAACAGTATACCCTGCCGGTG
LinLab11328/EEB	Delete *MOB2* gRNA-scaffold forward	TGCTGAGTGGGAGTGTTGGTGTTTTAGAGCTAGAAATAGCAAGTT
LinLab11323/EEB	Amplify *MOB2* deletion cassette forward	GTTGGTACCTGACGGTCC
LinLab11324/EEB	Amplify *MOB2* deletion cassette reverse	ACGCTGTGCGTTTCAGTC

Supporting Information References

1. Nielsen K, Cox GM, Wang P, Toffaletti DL, Perfect JR, Heitman J. 2003. Sexual cycle of *Cryptococcus neoformans* var. *grubii* and virulence of congenic a and α isolates. Infect Immun 71:4831-4841.
2. Chadwick BJ, Pham T, Xie X, Ristow LC, Krysan DJ, Lin X. 2022. The RAM signaling pathway links morphology, thermotolerance, and CO_2 tolerance in the global fungal pathogen *Cryptococcus neoformans*. Elife 11.
3. Liu OW, Chun CD, Chow ED, Chen C, Madhani HD, Noble SM. 2008. Systematic genetic analysis of virulence in the human fungal pathogen *Cryptococcus neoformans*. Cell 135:174-188.
4. Walton FJ, Heitman J, Idnurm A. 2006. Conserved elements of the RAM signaling pathway establish cell polarity in the basidiomycete *Cryptococcus neoformans* in a divergent fashion from other fungi. Mol Biol Cell 17:3768-3780.
5. Shi R, Lin X. 2024. Illuminating the *Cryptococcus neoformans* species complex: unveiling intracellular structures with fluorescent-protein-based markers. Genetics 227:iyae059.
